# Combining ability, heritability and genotypic relations of different physiological traits in cacao hybrids

**DOI:** 10.1371/journal.pone.0178790

**Published:** 2017-06-19

**Authors:** Allan Silva Pereira, Alex-Alan Furtado de Almeida, Márcia Christina da Silva Branco, Marcio Gilberto Cardoso Costa, Dario Ahnert

**Affiliations:** 1 Departamento de Ciências Biológicas, Universidade Estadual de Santa Cruz, Campus Soane Nazaré de Andrade, Rod. Jorge Amado, Ilhéus, BA, Brasil; 2 Centro de Pesquisas do Cacau, Comissão Executiva do Plano da Lavoura Cacaueira (CEPEC/CEPLAC). Rod. Jorge Amado, Ilhéus, BA, Brasil; United States Department of Agriculture, UNITED STATES

## Abstract

Selecting parents and evaluating progenies is a very important step in breeding programs and involves approaches such as understanding the initial stages of growth and characterizing the variability among genotypes for different parameters, such as physiological, growth, biomass partitioning and nutrient translocation to the aerial part. In these cases, facilitating tools can be used to understand the involved gene dynamics, such as diallel crosses and genetic and phenotypic correlations. Our main hypothesis is that the contrasting phenotypes of these parental genotypes of cocoa used are due to genetic factors, and progenies derived from crosses of these parental genotypes are useful for breeding programs related to plant architecture, physiological parameters and translocation of mineral nutrients. We aimed to evaluate the combining abilities in progenies of cacao (*Theobroma cacao* L) originating from contrasting parents for canopy vigor. Emphasis was given to the evaluation of morphological and physiological parameters and the phenotypic and genotypic correlations to understand the dynamics of the action of the genes involved, as well as in expression profile from genes of gibberellins biosynthesis pathway in the parents. Fifteen *F1* progenies were obtained from crosses of six clones (*IMC 67*, *P4B*, *PUCALA*, *SCA 6*, *SCA 24* and *SJ 02*) that were evaluated in a randomized complete block design with four replicates of 12 plants per progeny, in a balanced half table diallel scheme. It is possible to identify and select plants and progenies of low, medium and high height, as there is expressive genetic variability for the evaluated parameters, some of these on higher additive effects, others on larger nonadditive effects and others under a balance of these effects. Most physiological parameters evaluated show that for selection of plants with the desired performance, no complex breeding methods would be necessary due to the high and medium heritability observed. Strong genetic components were observed from many of the correlations, which indicate the possibility to formulate selection indices for multi-traits, such as dwarfism or semidwarfism, tolerance to increase of leaf sodium concentrations and maintenance of the photosynthetic apparatus integrity under these conditions. Additionally, plants with higher carbon fixation, better water use, higher carboxylation efficiency and greater magnesium accumulation in leaves can be selected.

## Introduction

Cacao seeds are an important agricultural commodity produced in more than 20 countries in the tropical regions of *A*frica, South and Central *A*merica and *A*sia. The main producers are Cote d'Ivoire (33%), Indonesia (18.7%), Ghana (17.6%), Nigeria (7.66%), Cameroon (5.12%) and Brazil (5.06%), and both marketing and consumption involves many countries around the world [1].

Although Brazil is among the world's leading cacao producers, the Brazilian yield per planted area is low, given the productivity potential of the crop. In Brazil and in the world, productivity problems are usually a reflection of the susceptibility to biotic and abiotic factors, such as disordered conformation of the plant architecture (unfavorable to planting densities due to self-shading), water status, nutrition, diseases and pests [2–8].

Cacao plants (*Theobroma cacao* L.) are usually propagated from seeds or different types of clonal propagation. When the plants are from seeds, the initial growth is orthotropic (formation of the main support structure of the crown, first order stem), and, at this stage, the phenotypic expression is the response of endogenous controls rather than from environmental stimuli. Subsequently, the growth is plagiotropic, whose onset is characterized by branching after the end of the orthogonal growth [3].

Selecting parents and evaluating progenies is a very important step in breeding programs. Mistaken selection compromises the potential of improved products/cultivars and, consequently, the demands of the program would not be met. Understanding the initial growth stages, as well as characterizing the variability between genotypes for physiological, growth, biomass partitioning and nutrient translocation to the aerial part parameters, as well as genetic and phenotypic correlations between these parameters are helpful approaches for selection of plants with traits of interest [9–12]. A tool of great utility in selection is the evaluation of parentals in diallel crosses schemes, with analyzes related to combining abilities in plant species that allow selecting the best parents and predicting hybrids [13]. Analyses of the diallel scheme have been used for evaluation of different agronomic and productivity [14,15], use and translocation of nutrient [16], physiological [17], content of chemical compounds [18], abiotic stresses [19,20] and morphological [21] parameters. One of the aspects of morphological parameter studies is the determination of sources of dwarfism and semidwarfism. The aim is to obtain high assimilate partitioning and agronomically adequate plants to be used in planting systems with high population density and high productivity [22,23].

In cacao, an approach to genetic mechanisms related to growth, biochemical and metabolic processes, and translocation of nutrients to leaves in progenies from contrasting parents for crown and root vigor, may be useful in targeting genetic improvement strategies for size reduction and increase of population density. These analyzes allow us to understand the dynamics of the genes involved in these parameters and the predominant type of effects (additives, non-additives or a balance of these effects) [24].

Our main hypothesis is that the parents with contrasting phenotype have genetic variability for the generation of progenies for breeding programs related to plant architecture, as well as for physiological parameters and translocation of mineral nutrients to the leaves. In order to test this hypothesis, we believe that the initial orthotropic growth phase is the most favorable, since cacao trees are less subject to external factors [3]. In this way, we aim to evaluate the combining abilities in progenies of cacao trees originated from contrasting parents for canopy vigor. *E*mphasis was given on the evaluation of morphological and physiological parameters and the phenotypic and genotypic correlations to understand the dynamics of the genes involved, as well as in expression profile from genes of gibberellins biosynthesis pathway in the parents.

## Material and methods

### Plant material

The experiment was conducted under greenhouse conditions at the Cacao Research Center, main research unit of the Commission of the Cacao Farming Plan (CEPEC/CEPLAC) (14° 47' S, 39°16' W and 55 m altitude) in Ilhéus, Bahia, Brazil, from 2013 to 2014. Plants of six contrasting cocoa genotypes for height (Table 1), from CEPLAC’s Germplasm *A*ctive Bank (BAG), were crossed to generate the experimental material with subsequent monitoring of the fertilized flowers and consequent formation, development and maturation of pods.

**Table 1:** Characterization of the parental genotypes used in progenies formation.

Clone	Origen	Group	Compatibility	Height	Wrinkled leaf
***IMC 67***	Peru	Forastero	*A*uto incompatible	Medium	No
***P4B***	-	-	-	Medium	No
***PUCALA***	Peru	Forastero	-	Low	Yes
***SCA 6***	Peru	Forastero	*A*uto incompatible	Low	No
***SCA 24***	Peru	Forastero	*A*uto incompatible	Low	No
***SJ 02***	Brazil	Trinitario	*A*uto compatible	High	No

Source: International Cacao Germplasm Database (ICGD), 2016.

From the crosses between the six genotypes, 15 hybrid cacao progenies were generated. From each progeny, five fruits were collected from which 48 randomly picked seeds were planted in 25 L pots containing soil. The plants were fertilized according crop recommendations [25]. The experiment was conducted in a randomized block design with four replications of 12 plants per progeny, in a diallel half table balanced scheme, with evaluation of the F1's hybrids (Table 2).

**Table 2:** Scheme of diallel half table, balanced, with 15 hybrid progenies.

PARENTAL	P4B	PUCALA	SCA 6	SCA 24	SJ 02
**IMC 67**	IMC 67 x P4B	IMC 67 x PUCALA	IMC 67 x SCA 6	IMC 67 x SCA 24	IMC 67 x SJ 02
**P4B**		P4B x PUCALA	P4B x SCA 6	P4B x SCA 24	P4B x SJ 02
**PUCALA**			PUCALA x SCA 6	PUCALA x SCA 24	PUCALA x SJ 02
**SCA 6**				SCA 6 x SCA 24	SCA 6 x SJ 02
**SCA 24**					SCA 24 x SJ 02

### Biometric parameters

Height of the orthotropic axis (*SH*), stem diameter (*SD*), total leaf area (*LA*), leaf number (*LN*), root (*RDB*), stem (*SDB*), leaf (*LDB*) and total (*TDB*) dry biomasses, leaf area ratio (*LA*/*TDB*; *LAR*) and specific leaf area (*LDB*/*LA*; *SLA*). The height of the orthotropic axis, from immediately above ground to its upper extremity, and the stem diameter of the plants were measured using ruler and caliper, respectively. Leaf area was estimated by the light attenuation photoelectric method using an automatic Li-3100 leaf area meter (LI-COR, Lincoln, NB, US*A*). To determine the dry biomass, the plant parts were packed in paper ba*gs* and placed in a forced circulation oven at 70°C until constant weight. With the dry mass of the various organs of the plant, it was also evaluated the partition of biomass in the whole plant and the root/shoot ratio.

### Gas exchanges and fluorescence emission

Measurements of net photosynthesis (*A*), stomatal conductance to water vapor (*gs*), transpiration (*E*) and internal and atmospheric CO_2_ concentration ratio (*Ci*/*Ca*) were taken between 08h00 and 12h00 in the second or third fully expanded mature leaf from the apex of the plant, using a Li-6400 portable photosynthesis system (LI-COR, Lincoln, NB, US*A*) equipped with a 6400-02B RedBlue artificial light. During the measurements, the irradiance was kept constant at 800 μmol photons m^-2^ s^-1^. The minimum time, preset for stabilization of the readin*gs* was 30 s and the maximum to save each reading was 60 s. The maximum coefficient of variation (CV) allowed to save each reading was 0.3%. In addition to photosynthetically active radiation, the temperature of the block (26°C) and the concentration of CO_2_ (380 μmol mol^-1^) inside the leaf chamber were kept constant. In addition, the intrinsic (*A*/*gs*; iWUE) and instantaneous (*A*/*E*; WUE) water use efficiencies, the gas to air vapor pressure deficit (*VPD*) (*gs*/*VPD*_*L*_) and the instantaneous carboxylation efficiency (*A*/*Ci*) were estimated.

Measurements of fluorescence emission of chlorophyll *a* were performed on the same leaves used for gas exchange measurements using a portable fluorometer (Pocket P*EA* Chlorophyll Fluorimeter—v 1.10—Hansatech Instruments, Norfolk, UK). The evaluations were performed between 09h00 and 10h00 after the leaves were kept for 30 min in the dark for reflection of incident solar radiation, decrease of leaf temperature and oxidation of the entire photosynthetic electron transport system using appropriate clips. *A*fter dark adaptation the leaves were exposed to a saturating light pulse (3500 μmol m^-2^ s^-1^, wavelength of 650 nm) for 1 s. Data of fluorescence emission signals were recorded in the Pocket P*EA* using specific software. *A*mong the parameters obtained, only the initial (*Fo*), maximum (*Fm*) and variable (*Fv*) fluorescences, the maximum photochemical efficiency of PS2 (*Fv/Fm*), the maximum efficiency of the photochemical process of PS2 and, or the potential photochemical activity (*Fv*/*Fo*), quantum thermal dissipation production within PS2 (*Fo*/*Fm*) and plant performance or vitality index (*PI Inst*) were evaluated.

### Macro and micro mineral nutrients

For the determination of leaf macro and micronutrient concentrations in plants of the 15 progenies, 200 mg of dry and ground tissue of each sample were submitted to nitroperchloric digestion (3:1) for the determination, by colorimetry (725 nm), of phosphorus (*P*). Leaf concentrations of potassium (*K*) and sodium (*Na*) were determined by flame emission photometry, calcium (*Ca*), copper (*Cu*), iron (*Fe*), magnesium (*Mg*), manganese (*Mn*), and zinc (*Zn*) by atomic absorption spectrophotometry [26]. For nitrogen (*N*) determinations 200 mg of dried and ground tissue of each sample were subjected to sulfosalicylic digestion according to the Kjeldahl method [27].

### Parental gene expression

Mature leaf samples of parental of cacao were collected by freezing in liquid nitrogen. After, samples were stored at −80°C, lyophilized and then stored at– 20°C. RNA extraction was realized using *RNAqueous Kit* (Ambion − Applied Biosystems) according to the manufacturer instructions. RNA samples were treated with *DNase I* (Thermo Scientific) at 37°C for 30 min and quality was assessed by 1% gel electrophoresis. First strand cDNA synthesis from RNA templates was performed using a recombinant polymerase *RevertAidTM H-Minus M- MuLV* (Thermo Scientific) in the presence of *Oligo d(T)*_*18*_ *primer*. Reactions were incubated at 42°C for 60 min followed by 70°C for 10 min. for reverse transcriptase inactivation. The final product of reverse transcription was subjected to quantification in spectrophotometer *NanoDrop 2000c UV-Vis Spectrophotometer* (Thermo Scientific) and, then, diluted to 50 ng.

Abundance of transcripts of genes encoding enzymes involved in two stages of gibberellin biosynthesis in cacao parental genotypes (SCA 6, SCA 24, PUCALA, P4B, IMC 67, and SJ 02) was examined using specific primers as described in Table 3. Quantitative PCR Real Time was performed in the *ABI 7500 Real-Time PCR System* (Applied Biosystems) using *Power SYBR Green PCR Master Mix* (Applied Biosystems) kit according to manufacturer instructions. Reaction mix consisted of 100 ng of single strand cDNA as template, 0.3 μM of each primer and an appropriate amount of *Power SYBR Green PCR Master Mix* (Applied Biosystems) to complete the final volume of 20 μL.

**Table 3:** Pairs of gene-specific primers used in Real Time qPCR analysis.

Gene	Locus / Data bank	Primer sequence	Ta (°C)	Amplicon size (bp)
***TcCPS***	Tc01_t027780 (Cocoa Genome Hub)	F—5' CGAACCCTGAACCACCTATC 3' R—5' ACCACTAACTCTGCTACTCCT 3'	56	143
***TcKS***	XM_007022679.1 (GenBank)	F—5' CCTTTCCATCTGTGTCCTCG 3' R—5' TATCTCCTCCTCTCCGTGC 3'	58	135
***TcKO***	Tc08_t001220 (Cocoa Genome Hub)	F—5' GCAGGTTCTCTTCAGGCG 3' R—5' TCTCCATCGTTCAGTCTCCA 3'	60	86
***TcKAO2***	Tc03_t023490 (Cocoa Genome Hub)	F—5' AAAGGCGAAGGAAGAGCAAG 3' R—5' ACAAGTCAGAAGGGAACCAC 3'	58	127

*TcCPS = Ent-copalyl diphosphate* synthase (EC 5.5.1.13), *TcKS = Ent*-kaurene *synthase* (EC 4.2.3.19), *TcKO = Ent*-kaurene *oxidase* (EC 1.14.13.78) and *TcKAO2 = Ent*-kaurenoic *acid oxidase 2* (EC 1.14.13.79).

Reactions were incubated at 50°C for 2 min., 95°C for 10 min. followed by 40 cycles at 95°C for 15 s, and 1 min at the annealing temperature of each primer (Table 3). Specificity of each primer pair was monitored after each reaction by a dissociation curve analysis in order to verify the melting temperature of each primer and its target sequence, as well as the possibility of formation of nonspecific products. All reactions were subjected to the same conditions of analysis. Fluorescence data were normalized by the signal of ROX passive reference to correct the fluorescence signal fluctuations due to variations in the volume or evaporation. Fluorescence curves were analyzed with the *SDS Software v1*.*4* (Applied Biosystems) and threshold cycle (C_T_) values were determined. Relative expression numbers were calculated for each gene of interest as fold change in relation to control sample (calibrator) using the 2^-ΔΔCt^ method [28] with GAPDH, polyubiquitin and actin gene as endogenous control [29]. Samples of cacao ‘Comum’ were used as calibrator since there was no applied treatment. Thus, use of an external calibrator enabled the calculation of basal levels of gene expression in all parentals evaluated. Moreover, cacao ‘Comum’ was the first variety to be introduced in the Bahia state and accounted for approximately 50% of the area cultivated for cacao in this state until 2003 [30]. As ‘Comum’ variety constitutes the genetic background of Bahia cacao plantations, cacao genotypes, in this state, share a similar narrow genetic base and presents a low genetic diversity [30].

### Statistical analyses

The biometric, physiological and nutritional data had their assumptions for ANOVA evaluated and met [31,32], and as the molecular data, subsequently, means were ranked by the Scott-Knott test at 5% probability. For combined ANOVA, progenies effects, as well as general and specific combining abilities for biometric, physiological and translocation characteristics of nutrients were analyzed according to Griffing [33] from the evaluation of hybrids, with the significance of the mean squares of the general and specific combining abilities given by the *F* test. The ranking for the individual effects of each parent and progeny on the respective values of the general and specific combination abilities, because they show negative and positive values, were based on the deviations method [34].

Phenotypic and genotypic correlations were established as follows [35]:

$$\begin{array}{c} r_{p}=TMP_{xy}/\sqrt{}\left(TMS_{x}\times TMS_{y}\right);\;r_{g}=\sigma_{gxy}/\sqrt{}\left(\sigma_{gx}{}^{2}\times \sigma_{gy}{}^{2}\right)\\ \sigma_{gxy}=\left(TMP_{xy}\;\text{-}\;RMP_{xy}\right)/r;\;\sigma_{gx}=\left(TMS_{x}\;\text{-}\;RMS_{x}\right)/r\;and\;\sigma_{gy}=\left(TMS_{y}\;\text{-}\;RMS_{y}\right)/r \end{array}$$

Where TMP_*xy*_ is the mean product related to treatments effects for *x* and *y* traits; TMS is the mean squared related to treatments effects for *x* and *y* traits; σ_*gxy*_ is the estimated genotypic covariance for *x* and *y* traits; RMP_*xy*_ is the mean product related to residue effects for *x* and y traits; σ^2^_*gx* and_ σ^2^_*gy*_ are the estimated genotypic covariance for *x* and *y* traits and RMS is the product related to residue for *x* and *y* traits. Phenotypic and genotypic correlations, classified as very strong (0.90 to 1.0 or—0.90 to—1.0), strong (0.70 to 0.90 or—0.70 to—0.90), moderate (0.50 to 0.70 or—0.50) - 0.70), weak (0.30 to 0.50 or -0.30 to -0.50) and negligible (0.0 to 0.30 or 0.0 to—0.30) [36] were also evaluated for significance by the *t* test.

## Results

### Combination capacity and heritability

Initially, individual variance analyzes showed statistically different allometries for the parameters related to growth and biomass partitioning among progenies. The ranking of averages (Scott-Knott, p<0.05) is shown in S1 Appendix, accompanied by the amplitudes of variation between the most contrasting plants in the experiment and the corresponding coefficients of variation of each parameter. In the joint analyzes of variance among progenies, the mean squares of progenies showed statistically significant differences by the *F* test (p<0.01) for all determined parameters (Table 4).

**Table 4:** ANOVA of combining ability for growth and biomass parameters.

SV		Mean Squares							
	DF	*SH*	*SD*	*LA*	*LN*	*RDB*	*SDB*	*LDB*	*TDB*
**Progenies**	14	859**	11.4**	6764889**	99.2**	104**	80.5**	106**	657**
**GCA**	5	635**	17.8**	10663529**	46.0**	95.0**	152**	164**	905**
**SCA**	9	984**	7.90**	4598977**	129**	108**	40.7**	74**	520**
**Residue**	42	4.57	0.42	142354	5.73	2.29	1.50	4.17	6.97
**SSgca (%)**	-	26.4	55.7	56.3	16.6	32.8	67.5	55.1	49.2
**SSsca (%)**	-	73.6	44.3	43.7	83.4	67.2	32.5	44.9	50.8
**h^2^**	-	99.5	95.6	97.1	93.2	97.1	98.4	95.2	98.8

Source of variation (SV), degree of freedom (DF), stem height (*SH*), stem diameter (*SD*), leaf area (*LA*), leaf number (*LN*), root (*RDB*), stem (*SDB*), leaf (*LDB*) and total (*TDB*) dry biomasses, general combining ability (GCA), specific combining ability (SCA), sum of squares of the general combining ability (SSgca), sum of squares of the specific combining ability (SSsca) and mean heritability (h^2^). P<0.05 (*) and two (**) asterisks indicate significance by *F* test at 5 and 1% probability, respectively.

**Fig 1:**
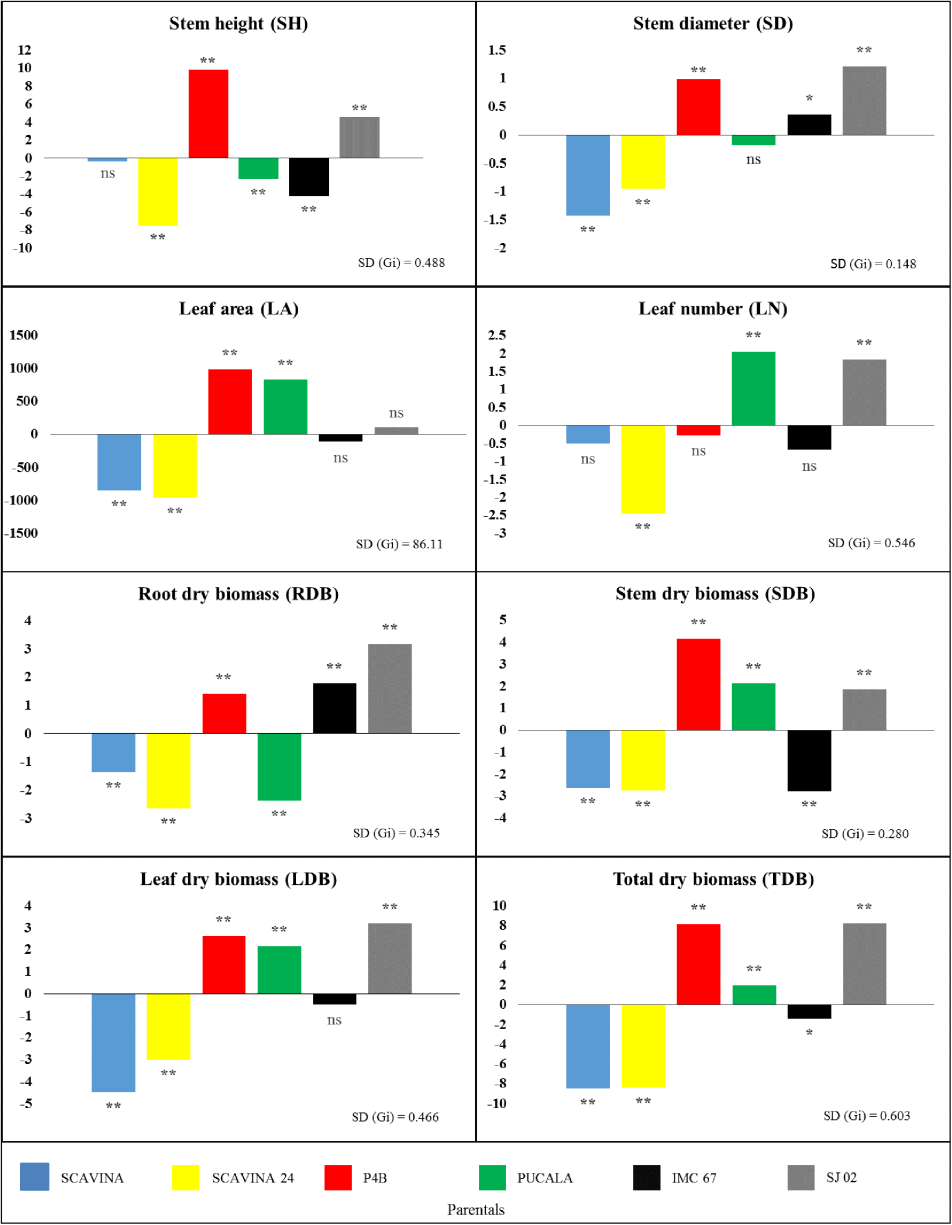
General combining ability (GCA) on parentals for growth and biomass parameters in cacao plants. Bars with 'ns' indicate absence of significance, * and ** denote GCA effects twice and thrice greater than SD (gi.), respectively.

When the mean squares of the evaluated parameters were decomposed in general (GCA) and specific (SCA) combination abilities, significant statistical differences were found by the *F* test (p<0.01) in both parameters. In order to understand the dynamics of the observed genetic variation, the percentages of the sums of squares of the GCA and SCA, which correspond to the total variation among progenies, were calculated (Table 4). The percentages of the sum of squares regarding GCA for *LDB*, *SD*, *LA* and *SDB* were higher when compared to SC*A*, ranging from 55.1 (*LDB*) to 67.5 (*SDB*). There was a balance between the percentages of the capacities (GCA = 49.2 and SCA = 50.8 for *TDB*). However, for most parameters, significant differences were observed between the percentages of SSgca and SSsca, which were higher for SCA. For *RDB*, *SH*, *LN* the percentages found were 67.2, 73.6 and 83.4, respectively. The mean heritability values in the broad sense for each characteristic were also evaluated and ranged from 93.2 to 99.5% for LN and SH, respectively. Therefore, there is considerable genetic influence on the evaluated parameters and little environmental influence, which signals for high possibilities of genetic gains in breeding programs. Concerning the GCA effects of each parent, the significant effects, which were observed for all parameters (Fig 1) showed contrasts of well-defined parents' influences.

*SJ 02* showed positive and significant GCA effects (p<0.01) for all parameters, except for *LA*, whereas *SCA 24* showed significant negative effects (p<0.01) for all parameters. Similar to SCA 24, *SCA 6* also had negative effects for all parameters but statistical significance (p<0.01) was observed only for *SD*, *LA*, *SDB* and *TDB*. For each variable, the results with the greatest deviations from the mean (positive or negative) and significant (p<0.01) were: *SH* (*P4B* = 9.85 and *SCA 24* = -7.56), *SD* (*SJ 02* = 1.21 and *SCA 6* = -1.43), *LA* (*P4B* = 986 and *SCA 24* = -962), *LN* (*PUCALA* = 2.06 and *SCA 24* = -2.44), *SDB* (*P4B* = 4.15 and *IMC 67* = -2.78), *LDB* (*SJ 02* = 3.30 and *SCA 6* = -4.49) and *TDB* (*SJ 02* = 8.24 and *SCA 6* = 8.48).

When we analyzed the SCA values of cacao progenies for the growth parameters, we found that they differed statistically from each other by significant deviations, both positive and negative, observed for the parameters evaluated (Table 5). The progeny averages and their ranking can be observed in S1 Appendix.

**Table 5:** Effects of specific combining ability (SC*A*) on 15 cacao progenies for growth and biomass parameters.

Progenies	Specific Combining *A*bility							
	*SH*	*SD*	*LA*	*LN*	*RDB*	*SDB*	*LDB*	*TDB*
***SCA 6* x *SCA 24***	22.2**	1.00**	1346**	7.39**	3.39**	2.04**	4.84**	10.3**
***SCA 6* x *P4B***	-18.7**	-1.05**	-962**	-5.57**	-7.74**	1.49**	-3.18**	-9.42**
***SCA 6* x *PUCALA***	1.56^ns^	0.26^ns^	194^ns^	-0.78^ns^	3.80**	0.87^ns^	0.52^ns^	5.19**
***SCA 6* x *IMC 67***	4.18**	0.06^ns^	12.1^ns^	-1.05^ns^	-1.92**	-1.41*	-1.50^ns^	-4.83**
***SCA 6* x *SJ 02***	-9.28**	-0.27^ns^	-590**	0.01^ns^	2.47**	-3.00**	-0.68^ns^	-1.20^ns^
***P4B* x *SCA 24***	5.02**	1.00**	65.6^ns^	0.74^ns^	2.02**	-1.24*	2.27*	3.06*
***PUCALA* x *SCA 24***	-29.2**	-2.61**	-1736**	-10.9**	-8.53**	-5.88**	-8.59**	-23.0**
**IMC67 x *SCA 24***	0.98^ns^	-0.75**	-611**	0.40^ns^	0.09^ns^	1.58**	0.19^ns^	1.86^ns^
***SJ 02* x *SCA 24***	0.94^ns^	1.50**	935**	2.38*	3.03**	3.50**	1.29^ns^	7.82**
***P4B* x *PUCALA***	10.0**	0.38^ns^	148^ns^	4.66^ns^	2.56**	0.12*	0.23^ns^	2.91*
***IMC 67* x *P4B***	-1.84*	0.81**	989**	-0.61^ns^	1.50*	-1.07*	0.08^ns^	0.51^ns^
***P4B* x *SJ 02***	5.45**	-1.01**	-240^ns^	0.78^ns^	1.65*	0.70^ns^	0.60^ns^	2.94*
**IMC67 x *PUCALA***	5.67**	1.04**	554**	5.73**	4.83**	3.50**	5.14**	13.5**
***PUCALA* x *SJ 02***	11.9**	0.94**	839**	1.30^ns^	-2.66**	1.40**	2.70**	1.44^ns^
***IMC 67* x *SJ 02***	-9.00**	-1.16**	-944**	-4.50**	-4.49**	-2.60**	-3.91**	-11.0**
**S.D. (gi)**	0.828	0.251	146.13	0.927	0.586	0.474	0.791	1.022
**S.D. (gi—gj)**	1.309	0.395	231.05	1.465	0.927	0.750	1.250	1.617

* and ** = significance when SCA effects were twice and thrice greater than S.D. (gi.), respectively. Stem height (SH), stem diameter (*SD*), leaf area (*LA*), leaf number (*LN*), root (*RDB*), stem (*SDB*), leaf (*LDB*) and total (*TDB*) dry biomasses.

The growth parameters *SH*, *SD*, *LA*, *LDB* and RDB formed four groups regarding statistical significance. *SH* and *LA* showed significant (p<0.01) and non-significant positive values and only significant negative values (p<0.05 and p<0.01) among progenies. Similar behavior was verified for *RDB* which showed positive non-significant and significant (p<0.05 and p<0.01) values and significant negative values (p<0.01). For *SD* and *LDB* positive and negative values with (p<0.05 and p<0.01) and without significance were determined. On the other hand, five groups were observed for *LN*, *SDB* and *TDB*. In this case, *LN* and *TDB* showed non-significant and significant positive (p<0.05 and p<0.01) values and negative non-significant and significant (p<0.05 and p<0.01) estimates. *SDB* showed non-significant and significant positive (p<0.05 and p<0.01) values and negative estimates at p<0.01 and p<0.05.

*SCA 6* x *SCA 24* and *IMC 67* x *PUCALA* crosses showed positive and significant (p<0.01) estimates for all parameters. The observed effect in *IMC 67* x *PUCALA* was combined with the positive effect of GCA of one the parents for almost all parameters, except for *SH*, in which both parents had negative GCA. For the same trait, progenies of *IMC 67* x *SJ 02* and *PUCALA* x *SCA 24* showed significantly negative estimates for *SCA*, with corresponding negative GCA estimates for at least one of the parents, except for *SD* and *RDB*, whose parents *IMC 67* and *SJ 02* showed positive GCA (Table 5, Fig 1).

Hybrids with higher positive and significant (p<0.01) SC*A* for *SH* were *SCA 6* x *SCA 24* (22.2), *PUCALA* x *SJ 02* (11.9) and *P4B* x *PUCALA* (10.0). The last two crosses have one of the parents (*P4B* and *SJ 02*) with high GCA values. The hybrids *PUCALA* x *SCA 24*, *SCA 6* x *P4B*, *SCA 6* x *SJ 02* and *IMC 67* x *SJ 02* showed highly significant negative SC*A* values (-29.2, -18.7, -9.28 and -9.00, respectively), that is, they did not show favorable SCA for the increase of *SH*. The two parents that formed the hybrid *PUCALA* x *SCA 24* showed significant negative GCA. The resulting progeny had the highest negative SC*A* for *SH* among the hybrids, maintaining the parents characteristic. This feature is promising for selection of small plants aiming increases in plant density (Table 5, Fig 1).

### Gas exchange and fluorescence

Mean squares for the effects of progenies/treatments for physiological parameters, evaluated from diallel variance analyzes, showed statistical differences among them by the *F* test (p<0.01), except for *Fo/Fm*, which did not show statistically significant differences (Table 6).

**Table 6:** ANOVA of combining ability for gas exchange and chlorophyll fluorescence parameters.

**SV**		**Mean Square**							
	**DF**	***A***	***Gs***	***Ci***	***E***	***Vpd***_***L***_	***WUE***	***iWUE***	***Ci/Ca***
**Progeny**	14	0.868**	0.0010**	32303**	0.131**	1.117**	8.71**	3703**	0.098**
**GCA**	5	0.942**	0.0019**	67623**	0.252**	2.407**	13.4**	9064**	0.196**
**SCA**	9	0.827**	0.0005**	12680**	0.064**	0.401**	6.11*	725^ns^	0.043**
**Residue**	42	0.15	0.0001	203	0.013	0.002	2.54	664	0.002
**SSgca (%)**	-	38.8	67.5	74.8	68.7	76.9	54.9	87.4	71.6
**SSsca (%)**	-	61.2	32.5	25.2	31.3	23.1	45.1	12.6	28.4
**h^2^**	-	93.1	98.7	99.7	95.5	99.9	86.7	91.4	99.1
**SV**		**Mean Square**							
	**DF**	***A/ci***	***Fo***	***Fm***	***Fv***	***Fo/Fm***	***Fv/Fm***	***PI inst***	***Fv/Fo***
**Progeny**	14	0.0003**	260678**	9777954**	4746184**	0.0001ns	0.00043**	0.663**	0.068**
**GCA**	5	0.0008**	397202**	19998692**	8965447**	0.0002ns	0.00036**	0.527**	0.059**
**SCA**	9	0.0001ns	184831**	4099767**	2402149**	0.0001ns	0.00047**	0.739**	0.074**
**Residue**	42	0.0001	7037	120157	157592	6.90635	0.00001	0.024	0.004
**SSgsa (%)**	-	87.1	54.4	73	67.5	50	29.7	28.4	30.9
**SSsca (%)**	-	12.9	45.6	27	32.5	50	70.3	71.6	69.1
**h^2^**	-	99.0	74.4	73.6	73.0	70.0	65.9	91.5	66.2

One (*) and two (**) asterisks indicate significance by the *F* test at 5 and 1% probability, respectively.

Source of variation (SV), degree of freedom (DF), net photosynthetic rate (*A*), stomatal conductance (*gs*), intercellular CO_2_ concentrations (*Ci*), transpiration rate (*E*), Vapor Pressure Deficit (*Vpd*_*L*_), instantaneous water-use efficiency (*WUE* = *A*/*E*), intrinsic water use efficiency (*iWUE* = *A*/*gs*), ratio of intercellular and atmospheric CO_2_ molar fraction (*Ci/Ca*), carboxylation efficiency *A/Ci*, initial chlorophyll fluorescence (*Fo*), maximal chlorophyll fluorescence (*Fm*), variable chlorophyll fluorescence (*Fm*−*Fo*) (*Fv*), maximal photochemical efficiency of PSII (*Fv/Fm*), maximum quantum yield of PS2 photochemistry (*Fv/Fo*), quantum yield baseline (*Fo/Fm*), performance index or plant vitality (*PI Inst*), general combining ability (GCA), specific combining ability (SC*A*), sum of squares of the general combining ability (SSgca), sum of squares of the specific combining ability (SSsca) and mean heritability (h^2^).

**Fig 2:**
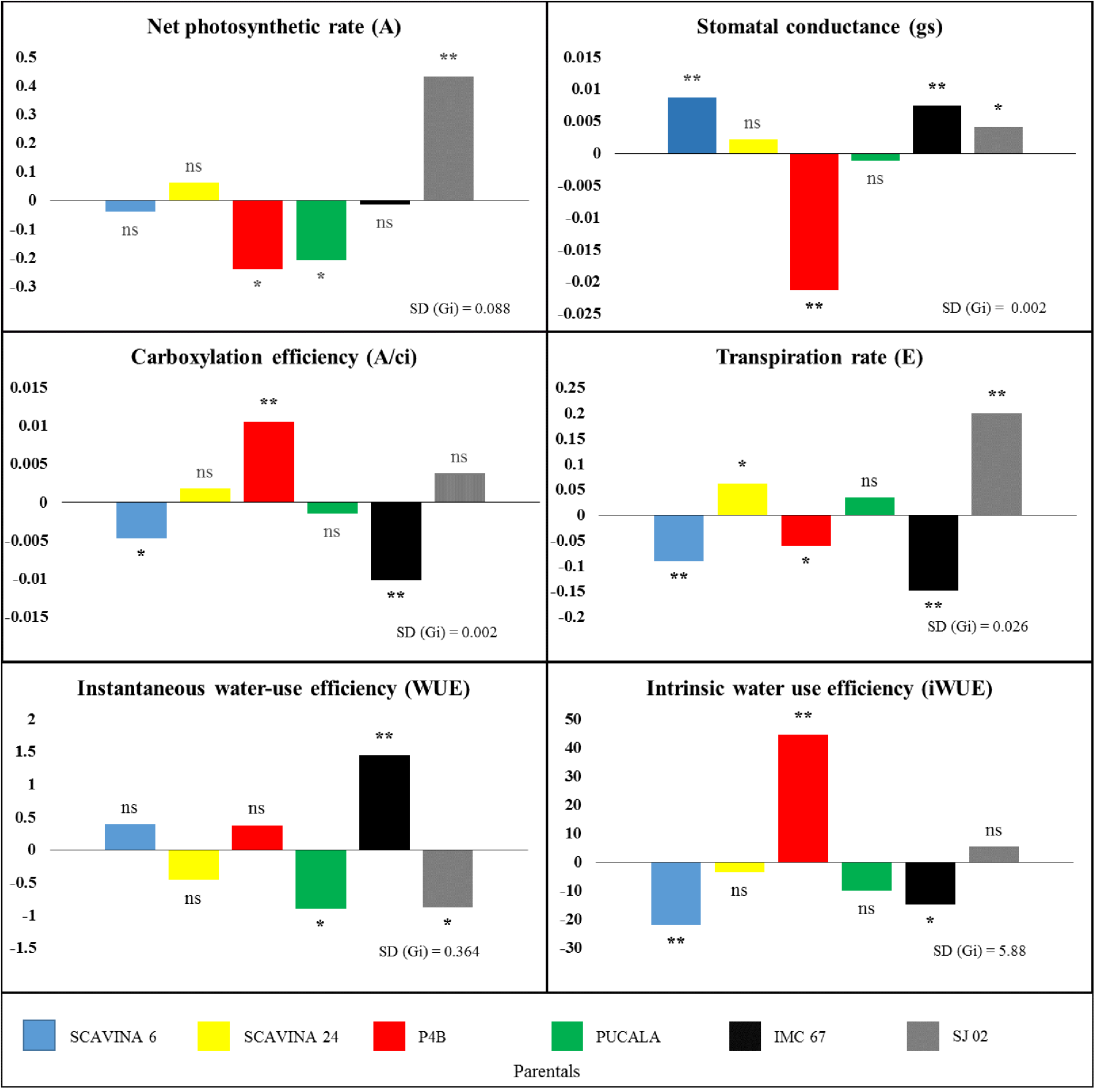
General combining ability (GCA) on parentals for gas exchange parameters in cacao leaves. Bars with 'ns' indicate absence of significance, * and ** denote GCA effects twice and thrice greater than SD (gi.), respectively.

For GCA and SCA, statistically different values were observed for almost all parameters by the *F* test (p<0.01), except for SCA for *WUE* (p<0.05), in addition to the non-significant SCA value of *iWUE* (Table 7, Fig 2), as well as GCA and SCA values for *Fo*/*Fm*. When we comparatively analyze the sums of squares of GCA and SCA, we observed higher values of SSgca for most of the parameters (*gs*, *Ci*, *VPDL*, *WUE*, *iWUE*, *Ci/Ca*, *A/Ci*, *Fo*, *Fm* and *Fv*), with a amplitude of 54.4% (*Fo*) to 87.4% (*iWUE*). On the other hand, the values of SSsca were higher for *A* (61.2%), *Fv*/*Fo* (69.1%) and *Fv/Fm* (70.3%). The mean heritability (h^2^) of the different physiological parameters showed values above 70% (Table 6).

**Table 7:** Effects of specific combining ability (SCA) on 15 cacao progenies for gas exchange and chlorophyll fluorescence parameters.

**Progenies**	***A***	***gs***	***Ci***	***E***	***Vpd***_***L***_	***WUE***	***iWUE***	***Ci/Ca***
***SCA 6* x *SCA 24***	0.08^ns^	0.004^ns^	49.1**	-0.079^ns^	-0.27**	1.24*	-0.96^ns^	0.09**
***SCA 6* x *P4B***	-0.39*	-0.010*	-92.5**	-0.006^ns^	0.49**	-1.03^ns^	-10.2^ns^	-0.19**
***SCA 6* x *PUCALA***	-0.14^ns^	-0.009*	36.8**	-0.111*	-0.32**	1.03^ns^	13.4^ns^	0.05*
***SCA 6* x *IMC 67***	-0.25^ns^	-0.008*	-61.6**	0.082^ns^	0.41**	-1.40*	7.54^ns^	-0.07**
***SCA 6* x *SJ 02***	0.70**	0.023**	68.2**	0.114*	-0.31**	0.15^ns^	-9.84^ns^	0.12**
***P4B* x *SCA 24***	0.19^ns^	0.001^ns^	12.0*	0.002^ns^	-0.04*	-0.24^ns^	13.0^ns^	0.02^ns^
***PUCALA* x *SCA 24***	0.11^ns^	0.004^ns^	-22.4**	0.107*	0.17**	-0.31^ns^	-0.99^ns^	-0.01^ns^
**IMC67 x *SCA 24***	0.25^ns^	0.004^ns^	-9.54^ns^	-0.041^ns^	-0.06**	0.26^ns^	0.24^ns^	-0.06**
***SJ 02* x *SCA 24***	-0.63**	-0.013**	-29.2**	0.012^ns^	0.20**	-0.95^ns^	-11.3^ns^	-0.04*
***P4B* x *PUCALA***	-0.36*	0.0003^ns^	8.12^ns^	-0.051^ns^	-0.07**	-0.61^ns^	-17.4^ns^	0.01^ns^
***IMC 67* x *P4B***	0.28^ns^	0.002^ns^	58.6**	-0.079^ns^	-0.30**	2.12**	-7.96^ns^	0.13**
***P4B* x *SJ 02***	0.29^ns^	0.007^ns^	13.8*	0.134**	-0.08**	-0.24^ns^	22.5*	0.03ns
**IMC67 x *PUCALA***	0.24^ns^	0.012**	21.4**	0.177**	-0.01^ns^	-1.07^ns^	3.22^ns^	0.04*
***PUCALA* x *SJ 02***	0.16^ns^	-0.007^ns^	-43.9**	-0.121*	0.23**	0.96^ns^	1.74^ns^	-0.08**
***IMC 67* x *SJ 02***	-0.52**	-0.010*	-8.91^ns^	-0.139**	-0.04*	0.08^ns^	-3.04^ns^	-0.04*
***SD* (gij)**	0.150	0.004	5.52	0.044	0.02	0.62	9.98	0.02
***SD* (gi—gj)**	0.237	0.006	8.72	0.070	0.03	0.98	15.8	0.03
**Progenies**	***A/Ci***	***Fo***	***Fm***	***Fv***	***Fo/Fm***	***Fv/Fm***	***PI inst***	***Fv/Fo***
***SCA 6* x *SCA 24***	-0.005^ns^	207**	549**	420*	0.001^ns^	0.005**	0.322**	-0.077**
***SCA 6* x *P4B***	0.008*	-186**	-15.8^ns^	162^ns^	-0.004^ns^	0.002*	-0.201**	0.141**
***SCA 6* x *PUCALA***	-0.004^ns^	202**	578**	361*	0.002^ns^	-0.005**	-0.384**	-0.070*
***SCA 6* x *IMC 67***	0.005^ns^	-79.2*	-1682**	-1401**	0.007^ns^	-0.001^ns^	-0.126*	-0.140**
***SCA 6* x *SJ 02***	-0.005^ns^	-143**	571**	458*	-0.006^ns^	-0.002*	0.389**	0.146**
***P4B* x *SCA 24***	0.004^ns^	-145**	163^ns^	-38.9^ns^	-0.004^ns^	-0.007**	0.127*	0.071*
***PUCALA* x *SCA 24***	0.002^ns^	-297**	-562**	-659**	-0.005^ns^	-0.007**	0.384**	0.091**
**IMC67 x *SCA 24***	0.002^ns^	111**	851**	1081**	-0.001^ns^	0.008**	-0.209**	0.071*
***SJ 02* x *SCA 24***	-0.003^ns^	124**	-1000**	-803**	0.009^ns^	0.0002^ns^	-0.624**	-0.16**
***P4B* x *PUCALA***	-0.008*	-26.2^ns^	-1231**	-203^ns^	0.006^ns^	0.023**	0.182**	-0.012^ns^
***IMC 67* x *P4B***	-0.008*	150**	875**	379*	-0.001^ns^	-0.009**	-0.331**	-0.032^ns^
***P4B* x *SJ 02***	0.004^ns^	207**	209^ns^	-299^ns^	0.004^ns^	-0.011**	0.224**	-0.167**
**IMC67 x *PUCALA***	0.003^ns^	63.9^ns^	476**	-101^ns^	-0.001^ns^	-0.011**	0.237**	-0.042^ns^
***PUCALA* x *SJ 02***	0.006^ns^	57.9^ns^	740**	603**	-0.003^ns^	0^ns^	-0.419**	0.033^ns^
***IMC 67* x *SJ 02***	-0.002^ns^	-246**	-519**	41.7^ns^	-0.004^ns^	0.012**	0.429**	0.143**
***SD* (gij)**	0.004	32.5	134.3	154	1.018	0.001	0.060	0.025
***SD* (gi—gj)**	0.006	51.4	212.3	243	1.610	0.002	0.095	0.040

* and ** = Significance when SCA effects were twice and thrice greater than SD (gi), respectively. Net photosynthetic rate (*A*), stomatal conductance (*gs*), intercellular CO_2_ concentrations (*Ci*), transpiration rate (*E*), Vapor Pressure Deficit (*Vpd*_*L*_), instantaneous water-use efficiency (*WUE* = *A*/*E*), intrinsic water use efficiency (*iWUE* = *A*/*gs*), ratio of intercellular and atmospheric CO_2_ molar fraction (*Ci/Ca*), carboxylation efficiency *A/Ci*, initial chlorophyll fluorescence (*Fo*), maximal chlorophyll fluorescence (*Fm*), variable chlorophyll fluorescence (*Fm*−*Fo* = *Fv*), maximal photochemical efficiency of PSII (*Fv/Fm*), maximum quantum yield of PS2 photochemistry (*Fv/Fo*), quantum yield baseline (*Fo/Fm*) e o performance index or plant vitality (*PI Inst*).

**Fig 3:**
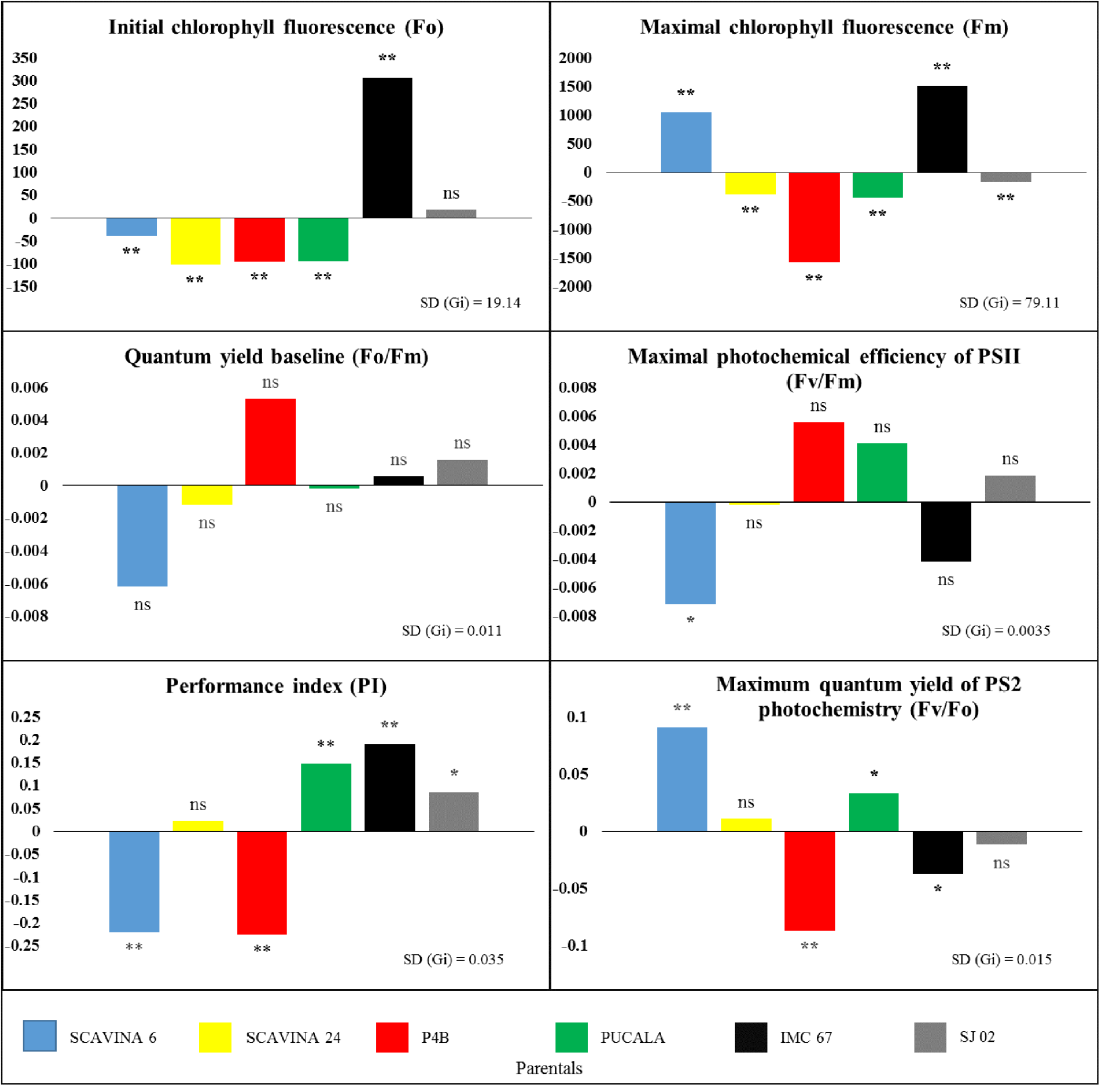
General combining ability (GCA) on parentals for chlorophyll fluorescence parameters in cacao leaves. Bars with 'ns' indicate absence of significance, * and ** denote GCA effects twice and thrice greater than SD (gi.), respectively.

The GCA estimates for the physiological parameters of each of the parents showed that at least one parent had a significant effect (p<0.01) on their performance. This corresponds to a magnitude of at least three times the standard deviation, except for *Fo/Fm* (Fig 3).

We observed that among the cacao parents there is variation in the arrangement of alleles for all parameters. For example, *IMC 67* showed the highest positive deviations from the mean (p<0.01) among the parents for the effects of *Ci* (not shown), *WUE*, *Ci*/*Ca* (not shown), *Fo*, *Fm*, *Fv* (not shown) and *PI*, with significance also for *gs*. On the other hand, it showed significant negative values for *E*, *VPD*_*L*_ (not shown), *iWUE*, *A*/*Ci* and *Fv/Fm*, besides no significant values for *A* and *Fo*/*Fm*. In contrast, *SJ 02* showed the highest positive deviations for *A* (0.434) and *E* (0.200) among the parents, with expressive value also for *VPD*_*L*_ (p<0.01), and positive effects for *Fv/Fm* and *PI inst* (p<0.05). *A*lso, *SJ 02* showed significant negative effects for *Ci*, *WUE* and *Fm* and not significant for the other parameters (Figs 2 and 3).

When we evaluated the effects of SCA of the progenies in the physiological traits, we observed differences in the progeny performance in relation to each parameters (Table 7).

In general, the results showed positive and negative deviations values, many of them significant with respect to the mean. The parameters *E*, *Ci*/*Ca*, *Fv/Fm* and *Fv*/*Fo* formed five groups each regarding significance (not significant and with negative and positive values at p<0.05 and p<0.01). With regard to *A*, *gs*, *VPD*_*L*_ and *Fo*, these parameters discriminated progenies in four groups (not significant and with negative values at p<0.05 and p<0.01, and positive with p<0.01). For the other characteristics, the following groups were formed: *Ci* and *Fv* (not significant and with negative values at p<0.01 and positives at p<0.05 and p<0.01), *WUE* (not significant and negative values at p<0.05 and positive at p<0.05 and p<0.01), *PI* (with negative values at p<0.05 and p<0.01, and positive to p<0.05 and p<0.01), *A*/*Ci* (no significant and with negative values at p<0.05 and positive to p<0.01) and *Fm* (no significant and with negative and positive at p<0.01) (Table 7).

### Mineral nutrients accumulation

When evaluating the mean squares of progenies/treatments effects for translocation of mineral nutrients, we observed statistically significant differences among progenies by the *F* test (p<0.01) for the translocation of all nutrients to the leaves Table 8).

**Table 8:** ANOVA of combining ability for mineral nutrient leaf content.

SV		Mean Square									
	DF	*P*	*K*	*Na*	*N*	*Ca*	*Mg*	*Fe*	*Zn*	*Cu*	*Mn*
**Progenies**	14	0.72**	72.5**	0.62**	16.8**	3.76**	2.22**	88756**	846**	20.9**	22995**
**GCA**	5	0.47**	118**	0.40**	21.7**	0.60^ns^	1.60**	125610**	222^ns^	33.4**	13123**
**SCA**	9	0.85**	47.0**	0.74**	14.1**	5.51**	2.56**	68281**	1193**	14.0*	28479**
**Residue**	42	0.12	8.34	0.11	1.79	0.60	0.26	5313	231	4.88	3218
**SSgca (%)**	-	23.3	58.3	23.2	46.1	5.7	25.7	50.5	9.4	57.0	20.4
**SSsca (%)**	-	76.7	41.7	76.8	53.9	94.3	74.3	49.5	90.6	43.0	79.6
**h^2^**	-	52.7	85.0	66.9	76.6	59.7	82.1	88.5	65.0	52.3	80.7

P<0.05 (*) and two (**) asterisks indicate significance by *F* test at 5 and 1% probability, respectively. Source of variation (SV), degree of freedom (DF), phosphorus (*P*), potassium (*K*), sodium (*Na*), nitrogen (*N*), calcium (*Ca*), magnesium (*Mg*), iron (*Fe*), zinc (*Zn*), copper (*Cu*) and manganese (*Mn*), general combining ability (GCA), specific combining ability (SC*A*), sum of squares of the general combining ability (SSgca), sum of squares of the specific combining ability (SSsca) and mean heritability (h^2^)

Based on these results the treatment effects were decomposed in GCA and SC*A*. We found that the GCA of the parents related to *P*, *K*, *Na*, *N*, *Mg*, *Fe*, *Cu* and *Mn* were statistically different from each other. In addition, the SC*A* observed in the progenies, for all nutrients, also showed significant statistical differences (p<0.01) by the *F* test. For the analyzes of the gene dynamics of the parameter variations, we observed, from the decomposition of the sums of the squares of progenies, that SSgca accounted for 58.3% and 57.0% for K and Cu, respectively, whereas for P, Na, N, Ca, Mg, Zn and Mn the SSsca was higher than GCA, with amplitude of 53.9 to 94.3% for N and Ca, respectively. In order to understand the possibility of selecting cacao progenitors with higher nutrient translocation potential, we observed that among all minerals, *K*, *N*, *Mg*, *Fe* and *Mn* showed the highest heritability values in the broad sense (h^2^), above 70% (Table 8).

**Fig 4:**
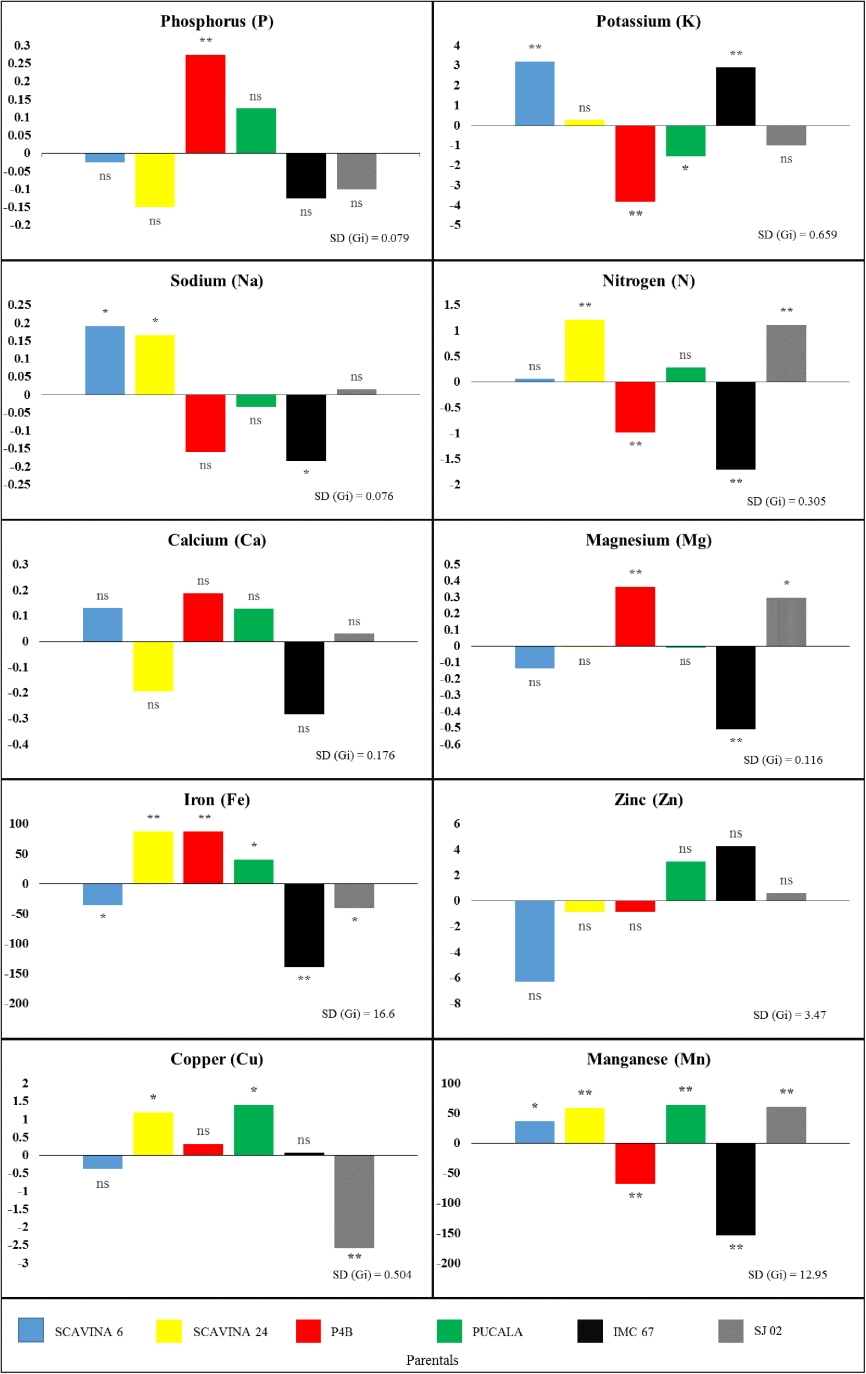
General combining ability (GCA) on parentals for mineral nutrient content in cacao leaves. Bars with 'ns' indicate absence of significance, * and ** denote GCA effects twice and thrice greater than SD (gi.), respectively.

When the performance of each parent is observed, from the GCA viewpoint, it is noticed that eight mineral nutrients showed statistical differences (p<0.05 and p<0.01) (Fig 4). It was found that for *P* accumulation, the *P4B* parent was statistically superior to the others (p<0.05), while for *K* accumulation the *SCA 6* and *IMC 67* parents were statistically superior (p<0.01). In addition, *SCA 6*, *IMC 67* and *SCA 24* accumulated more *Na* (p<0.01), whereas *SCA 24*, *SJ 02* and *P4B* accumulated more *N* in the leaves and were distinguished positively (p<0.05). In addition, *P4B* (p<0.01) and *SJ 02* (p<0.05) were superior from the other parents for *Mg*. Statistical differences were also showed for *Fe* by *SCA 24* and *P4B* (p<0.01) and *PUCALA* (p<0.05) in relation to the other four parents; *SCA 24* and *PUCALA* were significantly different (p<0.05) from the others for *Cu*; and *PUCALA*, *SJ 02* and *SCA 24* were statistically different for *Mn* (p<0.01) (Fig 4).

SCA estimates for leaf accumulation of practically all nutrients showed statistically different results (p<0.05 and p<0.01), except for *Ca* and *Cu* (Table 9). In descending order of quantities of formed groups, *Mn* was the only mineral that formed five distinct groups of progenies (not significance and with negative and positive values at p<0.05 and p<0.01). However, for *P*, *Na*, *Mg*, *Fe* and *Zn*, the progenies formed four groups (no significance and with negative and positive values at p<0.05 and p<0.01), whereas for *N* the progenies were grouped in no significant and with negative and positive values (p<0.05). In the case of *K* translocation, the progenies show groups with not significance and significant values (p<0.05), whereas for *Ca* and *Cu* no statistically significant differences were observed among progenies (Table 9).

**Table 9:** Effects of specific combining ability (SCA) on progenies for mineral nutrient content.

Progenies	*P*	*K*	*Na*	*N*	*Ca*	*Mg*	*Fe*	*Zn*	*Cu*	*Mn*
***SCA 6* x *SCA 24***	0.20^ns^	-3.20ns	-0.45*	-0.19ns	-0.45ns	0.39*	-158**	0.35ns	-0.42ns	7.12ns
***SCA 6* x *P4B***	0.07ns	-4.00ns	0.38ns	-2.19*	-0.66ns	-0.01ns	140**	-9.26ns	-2.14ns	-414**
***SCA 6* x *PUCALA***	0.02ns	0.23ns	-0.25ns	1.84*	0.15ns	-0.19ns	6.62ns	22.9*	-0.62ns	400**
***SCA 6* x *IMC 67***	0.17ns	4.88*	-0.40ns	1.34ns	0.83ns	0.85**	114*	-0.01ns	1.61ns	121*
***SCA 6* x *SJ 02***	-0.46*	2.08ns	0.71**	-0.79ns	0.13ns	-1.04**	-104*	-14.0ns	1.56ns	-114ns
***P4B* x *SCA 24***	-0.51*	4.03*	0.01ns	0.96ns	0.20ns	-0.54*	169**	-2.48ns	-0.22ns	190**
***PUCALA* x *SCA 24***	0.35ns	-0.35ns	0.48*	-0.02ns	-0.33ns	0.10ns	11.3ns	-20.1*	2.31ns	-248**
**IMC67 x *SCA 24***	-0.31ns	-1.70ns	0.13ns	-1.32ns	-1.42ns	-1.22**	-93.8*	-10.2ns	-0.87ns	43.1ns
***SJ 02* x *SCA 24***	0.27ns	1.21ns	-0.17ns	0.56ns	2.01ns	1.3**	71.1ns	32.5**	-0.82ns	7.74ns
***P4B* x *PUCALA***	0.02ns	3.96*	-0.10ns	1.99*	1.26ns	0.03ns	-111*	6.80ns	1.39ns	199**
***IMC 67* x *P4B***	0.67**	-2.10ns	0.16ns	-0.02ns	-0.06ns	0.26ns	-88.5*	9.67ns	1.81ns	-161*
***P4B* x *SJ 02***	-0.26ns	-1.90ns	-0.45*	-0.74ns	-0.74ns	0.26ns	-110*	-4.73ns	-0.84ns	186**
**IMC67 x *PUCALA***	-0.68**	-1.77ns	0.03ns	-2.39*	0.49ns	0.34ns	9.3ns	2.35ns	-2.87ns	-137*
***PUCALA* x *SJ 02***	0.30ns	-2.07ns	-0.17ns	-1.42ns	-1.57ns	-0.28ns	83.8*	-12.0ns	-0.22ns	-214**
***IMC 67* x *SJ 02***	0.15ns	0.68ns	0.08ns	2.39*	0.17ns	-0.29ns	58.6ns	-1.78ns	0.31ns	134*
***SD* (gi)**	0.21	1.90	0.22	0.88	1.83	0.19	40.4	9.41	1.46	59.2
***SD* (gi—gj)**	0.33	3.00	0.35	1.39	2.89	0.29	63.9	14.88	2.31	93.6

* and ** = significance when SCA effects were twice and thrice greater than S.D. (gi.), respectively. Phosphorus (*P*), potassium (*K*), sodium (*Na*), nitrogen (*N*), calcium (*Ca*), magnesium (*Mg*) iron (*Fe*) zinc (*Zn*), copper (*Cu*) e manganese (*Mn*).

At least one progeny was different from the others for translocation of mineral nutrients, with the exception of *Ca* and *Cu*, for which all progenies were statistically the same. Of the 15 progenies evaluated (Table 9), 12 had statistically superior SCA for translocation of at least one nutrient, with emphasis on *Mn*, for which five progenies were superior [*SCA 6* x *PUCALA*, *P4B* x *PUCALA*, *P4B* x *SCA 24*, *P4B* X *SJ 02* (p<0.01) and *IMC 67* x *SJ 02* (p<0.05)]. In a comparative analysis between GCA of the parents and SCA of the progenies, we found that for *P*, *K*, *Mg* and *Fe*, the respective progenies that differed themselves from the others had at least p<0.05 of the parents with the highest GCA (Fig 4).

**Fig 5:**
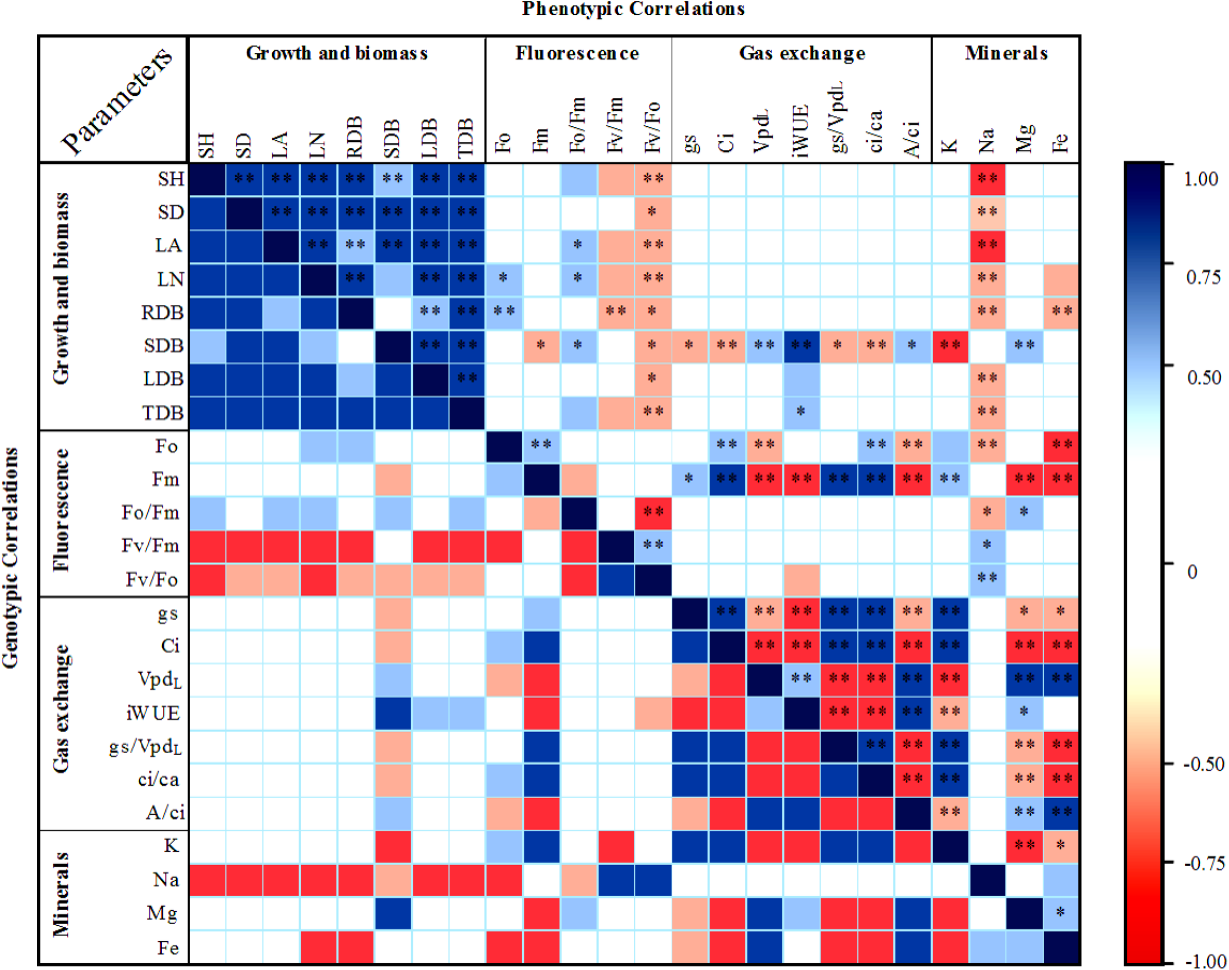
Phenotypic (above diagonal) and genotypic (below diagonal) correlation coefficients between physiological characteristics. In blue the positive correlations and in red the negative correlations with p<0.05 (*) and p<0.01 (**). The parameters with most significant correlations are shown. Low correlations between -0.5 and 0.5 are in blank. For all correlations, see S5 Appendix.

Phenotypic and genotypic correlations between the physiological parameters of gas exchange, chlorophyll fluorescence, growth and translocation of mineral nutrients at leaf level were also evaluated. *A* total of 528 estimates of phenotypic correlations and an equal number of genotypic correlations were obtained, of which the most expressive can be visualized in Fig 5.

Among all the phenotypic correlations evaluated, 28% were significant (p<0.01 or p<0.05) by the *t* test. The correlations between the most representative variables were grouped as follows: 71 moderate correlations (56.3% negative) and 68 strong correlations (61.8% positive). For the genotype correlations, there was an increase in strong correlations, a total of 99 correlations (51.5% negative), and a reduction of moderate correlations with a total of 45 correlations, of which 54.3% were positive (S5 Appendix).

### Correlations between parameters within groups

For most representative correlations, high similarity was observed between phenotypic and genotypic correlations, regarding direction (positive or negative) and in magnitude, with two groups of strong correlations, both for phenotypic and genotypic correlations (upper left and bottom right regions of Fig 5). These results stimulate the joint description of both types of correlation with punctual discriminations. In the evaluated progenies, strong positive correlations were observed among most biometric parameters, while the correlations among gas exchange parameters were very strong, strong and moderate, some positive and some negative (Fig 5).

Net photosynthetic rate (*A*) strongly correlated with *gs* (r = 0.8, p<0.01) and moderately with *gs*/*VPD*_*L*_ (r = 0.69, p<0.01). Correlation between *A* and *E* (r = 0.5) and *Ci*/*Ca* (r = 0.48) were not significant (Fig 5, S2 Appendix). The other parameters were grouped with respect to the correlation direction (positive or negative), so that when *gs*, *Ci*, *gs*/*VPD*_*L*_ and *Ci*/*Ca* were positive, the others were too and vice versa. Correlations between parameters of this group were strong and significant (p<0.01) by the *t* test. The same occurred between *VPD*_*L*_ and *iWUE* for direction, which magnitudes were moderate or strong, but all significant (p<0.01). Correlations of *A*/*Ci* with *Ci*, *Ci*/*Ca*, *gs* and *gs*/*VPD*_*L*_ were negative and significant (p<0.01) by the *t* test. *A*mong the parameters related to leaf gas exchange, genotypic correlations followed their phenotypic counterparts in magnitude, with slightly higher coefficients for some genotypic correlations when compared with phenotypic correlations (S5 Appendix).

When we evaluated the correlations between parameters associated with chlorophyll fluorescence, we observed strong and moderate correlations, both positive and negative. The initial parameters *Fo* and *Fm* showed positive and significant (p<0.01 by the *t* test) mean phenotypic correlation with each other and their corresponding positive mean genotypic correlation and of the same magnitude. The *Fo*/*Fm*, *Fv/Fm* and *Fv*/*Fo* ratios could be analyzed from the results of *Fo* and *Fm* and the observation of *Fv*, which together give a broad view of the leaf photochemical state, especially with respect to the indication of stresses [37]. Phenotypic correlations of *Fv*/*Fo* with *Fo*/*Fm*, and also of *Fv/Fm* with *Fo*/*Fm* were negative, with strong and weak magnitudes, respectively, while the correlation of *Fv*/*Fo* with *Fv/Fm* was positive and moderate (r = 0.6). Although all genotypic correlations of the fluorescence ratios maintained the same signal of their respective phenotypic correlations, the genotypic correlation of *Fv*/*Fo* with *Fv/Fm* had its magnitude increased from moderate to strong (r = 0.9) (Fig 5, S5 Appendix).

Among mineral nutrients *K*, *Mg* and *Fe* were the most phenotypically correlated. We observed negative *K* correlations, both with *Mg* (strong and significant, p <0.01) and *Fe* (moderate and significant, p<0.05); Magnesium and *Fe* correlated positively with each other (moderate and significant, p <0.05) (Fig 5, S5 Appendix). Also, *Na* showed a moderate and positive no significant correlation with *Fe* and moderate, negative and significant (p<0.05) with *Zn*. Nitrogen showed moderate and positive correlations with *Cu* (*ns* by the *t* test) and *Mn* (p <0.05 by the *t* test). Some correlations had their magnitudes increased, such as the genotypic correlation between *K* and *P*, which was moderate and negative, whereas its phenotypic counterpart was low and negative, as well as the correlation between *K* and *Fe*, which changed from a moderate negative (phenotypic) to a strong negative (genotypic).

### Correlations between parameters of different groups

Generally, when we evaluated the correlations of growth parameters with fluorescence parameters of chlorophyll *a*, it was observed moderate negative correlations of *Fv/Fo* and *Fv/Fm* with all growth parameters, with the exception of *RDB* that showed moderate correlation (r = 0.65). The *Fo/Fm* parameter correlated positively with growth parameters; most of these correlations were moderate. In the case of *Fo* and *Fm*, the former showed moderate positive correlations only for *LN* and *RDB* and the latter moderate negative correlation with *SDB* (r = - 0.61) (Fig 5, S5 Appendix).

On the other hand, among the correlation of growth parameters with parameters related to leaf gas exchange only *SDB* correlated with most (70%) gas exchange parameters, with moderate magnitudes and positive or negative values, except for *iWUE*, which showed a strong positive correlation with *SDB*. In addition, *iWUE* showed moderate correlations with most (62.5%) growth parameters. *A*nalyzing the correlations of growth parameters with mineral translocation parameters, *Na* presented moderate negative correlations with most them, besides strong negative correlations with *SH* and *LA*. On the other hand, *Fe* presented moderate and negative correlations with *LN* and *RDB*, whereas with *SDB* the correlations with *Mg* and *K* were positive moderate and strong negative, respectively (Fig 5, S5 Appendix).

Among the chlorophyll fluorescence parameters only *Fm* correlated with all leaf gas exchange parameters, with positive correlations of moderate and strong magnitudes, as well as strong negative correlations. *A*dditionally, *Fv/Fo* correlated negatively with *iWUE*, and the magnitude in this case was moderate. When we analyzed the correlations between fluorescence parameters and mineral translocation, we noticed higher positive or negative correlation values. It was observed that *K* correlated moderately and positively with *Fo* and *Fm*. The correlations of *Na* with *Fo* and *Fo*/*Fm* were of moderate and negative magnitude, and *Na* with *Fv/Fm* and *Fv*/*Fo* were positive and strong. Magnesium showed a strong negative correlation with *Fm* and a moderate positive correlation with *Fo*/*Fm*, whereas *Fe* was strongly correlated with *Fo* and *Fm*. When we evaluated the correlations between leaf gas exchange and mineral nutrient translocation parameters, the correlations of *K*, *Mg* and *Fe* with gas exchange parameters were moderate and strong. Genotypic correlations tended to be larger when compared to their phenotypic counterparts, even with changes in magnitude, such as the correlations of *K* with *iWUE* and *A*/*Ci*, which changed from moderate to strong (Fig 5).

### Parental gene expression profile

**Fig 6:**
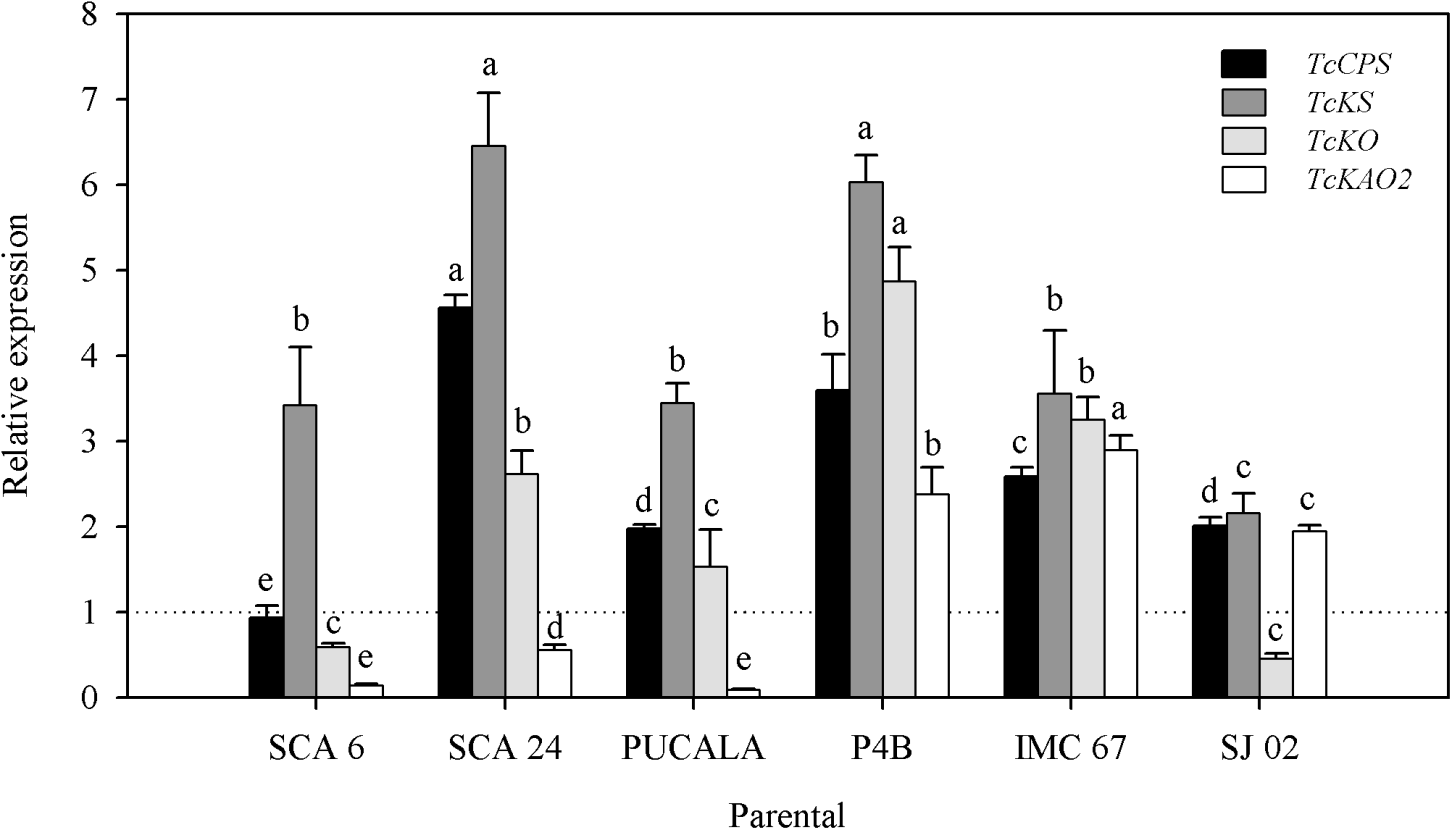
Relative quantification of expression of genes encoding enzymes involved in two stages of gibberellin biosynthesis. TcCPS—Ent-copalyl diphosphate synthase; TcKS—Ent-kaurene synthase; TcKO—Ent-kaurene oxidase; TcKAO2—Ent-kaurenoic acid oxidase 2, in leaves of six cacao parental genotypes (SCA 6, SCA 24, PUCALA, P4B, IMC 67 and SJ 02). Dotted line = calibrator. Multiple mean comparisons were made using Scott-Knott test (p < 0.05). Letters compare means among parentals for the same gene.

Relative expression of genes encoding enzymes involved in two stages of gibberellin biosynthesis was influenced significantly by effects of cacao parental genotypes (p < 0.01) (Fig 6).

Induction of expression of *TcCPS* was higher in leaves of SCA 24 followed by P4B, IMC 67 and SJ 02 (Fig 6) whereas in leaves of SCA 6 no change in expression was observed for *TcCPS*. Thus, the level of expression was equal to that of the calibrator. For TcKS gene, transcription was highly induced in leaves of SCA 24 and P4B followed by SCA 6, PUCALA and IMC 67 which shown the same level of expression. In contrast, a lower induction of *TcKS* transcription was observed in SJ 02 (Fig 6). Regarding the gene *TcKO*, leaves of P4B showed the highest transcription induction followed by IMC 67 and SCA 24. On the other hand, leaves of PUCALA showed non-significant induction of *TcKO* which did not differ significantly from repression level observed in SCA 6 and SJ 02. The *TcKAO2* gene showed higher induction in leaves of IMC 67 followed by P4B and SJ 02. In the other parentals genotypes, this gene was downregulated being the highest levels of repression observed in SCA 6 and PUCALA (Fig 6). When compared the TcKO and TcKAO2 expression levels, only in the SJ 02 parental genotype the TcKAO2 expression level was higher than TcKO expression level.

## Discussion

Currently, the main objectives of cacao breeding programs in the world are resistance to diseases [8,38,39], yield components [2], responses to abiotic stresses like drought stress and high light incidence and growth vigor [19,40] among other parameters. However, information on reduction of plant growth is lacking. In this work, we aim to provide information on the potential of cacao progenies, as well as their parents, regarding physiological parameters related to growth, gas exchange, chlorophyll fluorescence emission and translocation of minerals to leaves.

There was variation among the progenies/treatments for practically all the physiological parameters evaluated (Tables 4, 6 and 8 and S1, S2, S3 and S4 Appendixes). In practice, these results demonstrate a high variability among the progenies, which can be used in breeding programs for selection of parents and/or hybrids with the desired characteristics.

As for the dynamics of genes involved in the different physiological parameters, to better understand this variability, we followed the methodology described by Sprague and Tatum [41]. Thus, the relation between GCA and SC*A* indicates that for some parameters additive genic effects were more expressive (*LDB*, *SD*, *LA*, *SDB*, *gs*, *Ci*, *Vpd*_*L*_, *WUE*, *iWUE*, *Ci/Ca*, *A/Ci*, *Fo*, *Fm*, *Fv*, *K* and *Cu*), whereas for others non-additive effects (*RDB*, *SH*, *LN*, *A*, *Fv/Fo*, *Fv/Fm*, *P*, *Na*, *Ca*, *Mg*, *Zn* and *Mn*) and a balance between additive and non-additive gene effects (*TDB*, *Fo/Fm*, *N* and *Fe)* were more important (Tables 4, 6 and 8). The magnitudes of additive and non-additive gene effects show that both, selection among parents and within progenies and even a combination of these two strategies, would be promising for breeding. The significant allometries showed exhibit genetic consistency (h^2^ ≥ 70%), and, therefore, low difficulty in selection and possibility of considerable gains in breeding programs. (Tables 4, 6 and 8).

The amplitudes of variation for the growth parameters *SH*, *SD*, *LA*, *LN*, *RDB*, *SDB*, *LDB*, *TDB* and *LAR* (S1 Appendix) and the observed variability among progenies for the parameters involved in the canopy architecture (Table 4) mainly for *P4B* x *PUCALA* (high values of *SH*, *SD*, *LA* and increased of 31.6% on shoot biomass) and *PUCALA* x *SCA 24* (reduced *SH*, *SD*,*LA* and decrease of 34.7% on shoot biomass) emphasized the importance of understanding the crown architecture role in production and allocation of carbon in fruit trees as well as structures of source and sink, like is cited to apple trees, whose first-order stem is the main structural component of the canopy, besides being the place of fruit production, as occurs in cacao trees [42].

The phenotypic variability, with strong genetic components, observed among the cacao progenies for growth parameters and biomass allocation are promising for the delimitation of breeding strategies. *A*mong these strategies, the development of plants suitable for increasing the planting density is the main challenge. The choice of these strategies depends on what parameters one intends to improve. In this case, it would be interesting to improve on the basis of parents, but also to work the progeny variability, since SSsca for the above parameters, although smaller, were also expressive (Table 4). Other approach, for directional modifications of plant architecture, most of the observed variation for *SH*, *LN* and *RDB* were answered by the SSsca of each parameter in the progenies (Table 4). Thus, non-additive effects (types of dominance and, or epistatic) would be more important, with greater possibilities of gain and development of superior genotypes. So, for crown reduction, maybe the most promising expedient would be the combination of selection of individuals within progenies and also among different parents.

For growth analysis, crosses with SCAVINA (SCA 6 or SCA 24) showed the greatest influence on crown reduction when compared to the results of the other parents, since the progenies with the greatest potential for crown reduction have SCAVINA as their parental genotype. These results contrast with those observed by Padi and Colleagues [19]. They evaluated survival and vigor in different progenies of cacao under field conditions. Padi and Colleagues found that the progenies that had *SCAVINA* as their female parents were the most vigorous. However, when Bekele and Colleagues studied the morphological variations of cacao accessions at the International Germplasm Bank (IGB) in Trinidad, also described, the known *SCAVINA* accession (IGB code *SCA*), as well as a SC*A*VIN*A dwarf* (*SD*) [43]. Additionally, Efron and Colleagues selected a small, narrow-leaf mutant cacao plant in a progeny that had as one of the parents *SCA 12* [44].

In the present work, we can observe two evidences of the possibility of different alleles with dwarfing effects (with respect to the crown) in progenies that have *SCAVINA* as one of the parents. The first evidence is that their progenies showed a reduced crown phenotype (in relation to the experimental average), with influence in *SH* reduction. On the other hand, the cross between *SCA 6* and *SCA 24* showed medium-sized crown, but a combination of alleles that provided an increase of almost 15% in *SH* compared to the average. The second evidence is that when *SCAVINA* genotypes were crossed with *SJ 02* (high stature), which showed favorable alleles for crown growth, the two progenies presented distinct behavior regarding crown vigor. In *SCA 6* x *SJ 02* there was a decrease in height and crown biomass, while in *SCA 24* x *SJ 02* the crown vigor was average and the biomass increased. However, when the *SCAVINA* parents were crossed with *IMC 67*, which presented favorable alleles for reduction in size, both *SCA 6* x *IMC 67* and *IMC 67* x *SCA 24* showed parameters associated to reduced crown (S1 Appendix).

Some authors emphasizes the importance of reducing size and modeling plant architecture to avoid self-shading, by pruning [5]. Plants with reduced canopy vigor could meet or at least mitigate this demand, with the advantage of increasing the intervals between prunin*gs*, something economically advantageous. For PUCALA x SCA 24, which showed the most significant (p <0.01) negative SCA for reduction of components related to crown architecture (SH, SD, LA, SDB and LDB) (Table 5), a correspondence of negative and significant GCA was observed in both parents for some parameters and, in at least one parent, for others. This suggests, in turn, a significant allele concentration (p <0.01) for reduction of size in both parents (Fig 1). In addition, it is reported in the literature that different lines (such as the parents evaluated in the present experiment) may have different GCA-favorable alleles [45].

It was not unnoticed that *SCA 6* and *SCA 24*, which showed negative estimates of GCA, many of them significant (p <0.01) for all growth parameters (Fig 1), when combined in the *SCA 6* X *SCA 24* progeny, which presented positive and significant (p <0.01) SC*A* estimates for all growth parameters (Table 5, Fig 1). Possibly, alterations occurred in the allelic assemblages that conditioned reduction of size and, or biomass, and the group of alleles inherited by the progeny were favorable to increase size and biomass, such as *LDB*, *SD*, *LA* and *SDB*. For the parameters *RDB*, *SH* and *LN*, non-additive gene effects may explain the vigorous phenotype of *SCA 6* x *SCA 24*.

However, when *SCA 6* and *SCA 24* were crossbred with *SJ 02*, a genotype that showed positive and significant (p <0.01) GCA estimates for all growth parameters except for *LA*, the resulting progenies showed very different behaviors (Table 5, Fig 1). The allelic combinations in *SCA 6* x *SJ 02* were favorable for size and biomass reduction (negative SC*A*), whereas in *SCA 24* x *SJ 02* the allelic combinations were favorable for increasing size and biomass (SC*A* positive) (Table 5), demonstrating genetic effects on the phenotypes of these progenies. The same explanations apply to *PUCALA* x *SCA 24* and *SCA 6* x *SCA 24* progenies based on additive and non-additive gene effects. Changes in biomass allocation, for the development of dwarf genotypes, may alter biomass partitioning in favor of yield components [9]. The biomass partitioning patterns have been studied for decades in fruit trees [46] and the development of dwarf cocoa plants has been sought since the 1990s as a characteristic of agronomic quality [47].

Some authors studying annual crops report the importance of understanding how physiological parameters relate, as well as their combining abilities. These authors highlight the relationships between photosynthesis and plant growth and the accumulation of biomass [17]. In perennial crops such as cacao, understanding these relationships is also important, especially with regard to source and sink mechanisms, as well as understanding the flow of water from the leaf to the atmosphere, carbon from the atmosphere to the leaf and their reflections in photosynthate partitioning that influence the crown architecture formation [3,42]. These factors and their interactions are associated with light utilization efficiency, which variations constitute an interesting strategy for plant breeding [48].

The phenotypic amplitude for net photosynthetic rate (*A*) between plants (1–5.6 μmol de CO_2_ m^–2^ s^–1^) is consistent with results shown by other cacao reports [49,50]. The highest contrasts were observed between *SCA 6* x *SJ 02* (increase of 39.3% on average) and *P4B* x *PUCALA* (reduction of 22.7% on average). Genotypic variations observed among progenies about net photosynthesis (A), can be seen mainly on increase of A in SCA 6 x SJ 02 (Table 8) and the correspondence in the genitor SJ 02 (Table 6), with genic effects non-additive higher, but also with considerable additive effects (Table 5). In a comparative analyzes of different genotypes for gas exchanges, Daymond and contributors observed genotypic variations for A in IMC 67 and SCA 6, evaluated under the same developmental conditions [11]. As in that work, we observed in this experiment genotypic variations for *A*, where *gs* and *Ci* were responsible for the variation of *A* among progenies (S2 Appendix).

The range of phenotypic variation observed for *gs* (0.017 to 0.097 mol H_2_O m^–2^ s^–1^) (S2 Appendix) was as previously described by Almeida and contributors and Mielke and contributors [50,51], who found high variability in species of the Theobroma genus for the control of leaf gas-exchange with the external environment. Balasimha and contributors, evaluating leaf gas exchange of different genotypes also observed a genotypic effect in the observed variation for *gs* [12]. This can be reinforced from the determined *Ci*, *E* and *VPD*_*L*_ values. Progenies that showed higher *gs* values also showed higher *Ci* and *E*, and vice versa. However, progenies submitted to higher *VPD*_*L*_ also had lower *gs* values (S2 Appendix). For example, progenies of *P4B* x *PUCALA* and *PUCALA* x *SCA 24*, in *Vpd*_*L*_ of 2.1 kPa, had mean values of *gs* of 0.023 and 0.047 mol H_2_O m^–2^ s^–1^, respectively, whereas progenies *IMC 67* x *P4B* and *IMC 67* x *PUCALA*, at *Vpd*_*L*_ of 1.1 kPa, showed *gs* values of 0.034 and 0.064 mol H_2_O m^–2^ s^–1^, respectively (S2 Appendix). The decrease of *gs* may reduce CO_2_ fixation and its concentration in intracellular spaces [52]. Some authors reported a tendency to *gs* reduction with the increase in *VPD*_*L*_, although the relationship was not significant [49]. The influence of *VPD*_*L*_ on the reduction of *gs* is related to the response magnitude of each plant species [53]. In the present study, responses at the progeny level were quite different. The variability related to responses of the stomatal behavior to *VPD*_*L*_ among genotypes is reported in the literature [54].

The genotypic variations observed among progenies for net photosynthesis (*A*), can be seen mainly in the significant *A* increase (p <0.01) of *SCA 6* x *SJ 02* (Table 7) and significant correspondence (p <0, 01) in the genitor *SJ 02* (Table 6, Fig 2), with higher non-additive genic effects, but with considerable additive effects (Table 6). On the other hand, for *gs*, the SCA effects of *SCA 6* x *SJ 02* and *IMC 67* x *PUCALA* had correspondence in the GCA of *SCA 6* and *IMC 67* (p <0.01) (Table 7, Fig 2). However, for *Ci*, GCA values of *SCA 6* and *IMC 67* parents corresponded to positive and significant (p<0.01) SCA values of *SCA 6* x *SJ 02*, *IMC 67* x *P4B*, *SCA 6* x *SCA 24*, *SCA 6* x *PUCALA* and *IMC 67* x *PUCALA* progenies, in addition *P4B*, with negative and significant GCA (p<0.01), which corresponded to the significance of the negative effect of progeny *SCA 6* x *P4B*. As for *E*, the positive SC*A* values of *P4B* x *SJ 02* (p <0.01), *SCA 6* x *SJ 02* (p <0.05) and *PUCALA* x *SCA 24* (p <0.05) corresponded with the GCA of *SJ 02* (p<0.01) and *SCA 24* (p<0.05), whereas of the progenies with significant negative effects only *IMC 67* x *SJ 02* (p <0.01) corresponded with its genitor *IMC 67* (p<0.01) (Fig 2, Table 7). For these underlying parameters to gas exchange mentioned above, the major genic effects were additive, but with considerable non-additive effects (*gs* = 32.5%, *Ci* = 25.2% and *E* = 31.3%) (Table 5), which shows the great importance of parental selection, but also the possibility of considering progeny variations in selection stages of breeding programs.

Besides influencing CO_2_ assimilation, changes in *gs* also influence the control of water loss during transpiration [55]. Genotypes that manage to maintain regular rates of photosynthesis associated with less water loss have an advantage in environments with irregular rain distribution. On this respect, the amplitudes of water use efficiency values, from 3.0 to 9.3 μmol CO_2_ mmol^-1^ H_2_O for *WUE* and 48.6 to 180.8 μmol CO_2_ mol^-1^ H_2_O for *iWUE* (S2 Appendix) demonstrated variability among progenies likely to be used in breeding programs. To be clearer, regarding *WUE*, *IMC 67* x *P4B* was 45% above the mean, while *SJ 02* x *SCA 24* had 43.8% less *WUE* when compared to the experimental average. *A*s for *iWUE*, progenies *P4B* x *PUCALA* (62.5%) and *P4B* x *SJ 02* (62.1%) were more efficient, whereas *SCA 6* x *SJ 02* was 35.6% less efficient, compared to the experimental average (S2 Appendix). The variations found for *WUE* and *iWUE*, attributed to progenies effects, are in agreement with studies conducted by Daymond and colleagues for different cacao genotypes [11].

For *WUE*, the best result was observed for *IMC 67* x *P4B*, which showed the highest mean [8.3 μmol (CO_2_) mmol^-1^ (H_2_O)] (S2 Appendix) and a greater effect of SCA, which corresponded to GCA of the parent *IMC 67*, with both effects significant (p<0.01) (Fig 2, Table 7). For *iWUE*, the progeny *P4B* x *SJ 02* showed the highest SCA value (p<0.05), with GCA correspondence in the *P4B* parent (p<0.01) (Fig 2, Table 7). There was a slight superiority of the additive effects for *WUE* (54.9%), demonstrating that non-additive effects cannot be disregarded. On the other hand, the additive effects (87.4%) predominated with respect to *iWUE*, indicating the importance of selecting the parents for the design of genetic improvement programs to optimize the use of water resources (Table 6).

The efficiency of carboxylation (*A*/*Ci*), associated with the processing speed of fixed CO_2_, allows the study of factors subsequent to the stomatal cavities that may influence photosynthesis [56], and statistically discriminated progenies in the present work. The most efficient progenies with respect to *A*/*Ci* were *P4B* x *SCA 24*, *SCA 6* x *P4B* and *P4B* x *SJ 02* whereas the less efficient progenies were *IMC 67* x *PUCALA*, 6 x *IMC 67*, 6 x *PUCALA* and *IMC 67* x *P4B* (S2 Appendix). Under the conditions of the present experiment, if we consider the most contrasting progenies for *A*/*Ci*, and draw a parallel with *A* values of the same progenies, we can observe that *SCA 6* x *SJ 02*showed low *A/Ci* (0.013 mol m^–2^ s^–1^), high *A* (4.0 μmol (CO_2_) m^–2^ s^–1^) and high *Ci* = 305.7 μmol (CO_2_) mol^-1^. On the other hand, *P4B* x *SCA 24* showed high *A/Ci* (0.033 mol m^–2^ s^–1^), low *A* (3.2 μmol (CO_2_) m^–2^ s^–1^) and low *Ci (*113.2 μmol (CO_2_) mol^-1^) (S2 Appendix). This comparison may indicate that *gs* and *Ci* have a large influence on *A*/*Ci* and the observed variations may be partly a reflection of post-stomatal processes (non-stomatal constraints) related to Rubisco concentration, as well as their activity and, or regeneration rate [56,57]. Although, higher concentrations of *Ci* can cause inhibition of Rubisco oxygenase activity, competitively favoring the carboxylase activity and therefore increasing the photosynthetic rate [58]. Moreover, among the *A*/*Ci* results, *SCA 6* x *P4B* had its performance associated with the higher positive effects of SCA (p <0.05), which corresponded to the GCA effects of the *P4B* parent (p <0.01) (Fig 2, Table 6) and a predominance of additive effects (SSgca = 87.1%) (Table 5). However, directions in breeding programs for post-stomatal influences (such as activity, concentration and regeneration of Rubisco) would be more likely to be successful in selection among parents than among the progenies evaluated in the present work.

Correlations varied in the direct or inverse direction (+ or -). The net photosynthetic rate (*A*) was mainly influenced by *gs*, which can be verified by the strong positive and significant phenotypic correlation (r = 0.83; *t* test, p<0.01) and the corresponding genotypic correlation (r = 0.88) (Fig 5 and S5 Appendix) between these two factors, as has already been described in the literature [11]. The positive correlations of *gs* with *Ci*, *gs*/*VPD*_*L*_ and *Ci*/*Ca* show that the high *gs* values concurred for higher *Ci* values, as expected. Daley and contributors described a direct relationship between *gs* and *Ci* and conditioned the reduction of CO_2_ in the substomatic cavities and intercellular spaces to low *gs* rates [52]. The moderate negative correlation of *VPD*_*L*_ with *gs* (test *t*, p<0.01) showed that, under the conditions of the present experiment, a higher *VPD*_*L*_ is related to the reduction of *gs* and other related parameters (*Ci*, *Ci*/*Ca* and *gs*/*VPD*_*L*_), differing statistically from these, but did not have expressive influences on *A*. This can be verified by observing the correlation between *A* and *VPD*_*L*_ (S5 Appendix), which, although negative, was weak and not significant by the *t* test. Baligar and contributors reported reductions of *A* with increased *VPD*_*L*_. In parallel, these authors observed little reduction of *gs* and increase of *Ci*, both in response to the increase of *VPD*_*L*_, although it was not significant [49]. With the increase of *VPD*_*L*_, the plant tends to reduce *gs*, maintaining its water status, a survival strategy [59].

Regarding *WUE*, it presented a strong positive correlation with *Ci* and negative with *E* and *VPD*_*L*_, whereas *iWUE* showed strong negative correlations with *gs*, *Ci*, *Ci/Ca* and *gs/VPD*_*L*_, and positive with *A*/*Ci* (Fig 5). According to Massonnet and contributors, environmental factors such as temperature and relative humidity can influence *WUE* more directly, which may explain the negative correlation between *WUE* and *VPD*_*L*_. These authors report that *iWUE* show a more effective genetic control and it is less subjected to environmental factors than *WUE* [60]. Therefore, the negative correlations of *iWUE* with *gs*, *Ci*, *Ci/Ca* and *gs/VPD*_*L*_ are expected, since the greater efficiency of water use is related to the maintenance of internal CO_2_ concentrations that guarantee the maintenance of the plant physiological processes, without compromising water loss or increase. This is mainly achieved through stomatal control [50,61]. Significant genetic correlations between the parameters underlying gas exchange, such as correlations between *Ci/Ca* and iWUE, have been observed among different plant species [62]. Variability within a species is related to their ability to adjust to environmental variations, with a strong role of leaf biochemistry (such as *RUBISCO* carboxylation rate, electron transport rate) and stomatal responses [60].

Regarding carboxylation, its efficiency (*A*/*Ci*) showed strong positive and significant correlations (*t* test, p <0.01) with *VPD*_*L*_ and *iWUE*, strong negative correlations with *Ci*, *Ci/Ca* and *gs/VPD*_*L*_ and moderate negative correlation with *gs* (Fig 5). As previously mentioned, the increase of *VPD*_*L*_ was correlated with the decrease of *gs*. This limits the supply of new CO_2_ molecules into the stomatal cavity and favors increased carboxylation efficiency. Therefore, same-sense correlations, as of *A*/*Ci* with *gs* and *Ci* are expected. If that were not the case, reduction of *gs* and increase of *Ci* may reveal low carboxylation efficiency [56], which may be an important parameter in the study of variability between different specimens for the carboxylation efficiency of *RUBISCO*, as well as its concentration. As an example, we can compare the most contrasting progenies for *A*/*Ci* in the present work. In a joint analysis of *gs*, *Ci* and *A*/*Ci* values of *IMC 67* x *P4B* and *P4B* x *SCA 24* progenies, we can see that, with *gs* of 0.034 mol (H_2_O) m^-^^2^ s^-^^1^, the *IMC 67* x *P4B* progeny showed a *Ci* value of 268 μmol mol^-1^, whereas *P4B* x *SCA 24*, with *gs* of 0.030 mol (H_2_O) m^-^^2^ s^-^^1^ presented a *Ci* of 91.5 μmol mol^-1^. The *A/Ci* value for *IMC 67* x *P4B* was 0.01 mol (air) m^-2^ s^-1^, the mean *A/Ci* value for *P4B* x *SCA 24* was 0.041 mol (air) m^-2^ s^-1^ (S2 Appendix), with a slight numerical superiority in the *A* value for *P4B* x *SCA 24*, demonstrating post-stomatal effects on the differences between progenies.

The fluorescence emission of chlorophyll *a* parameters at the leaf level are considered a powerful tools to evaluate the efficiency of photosystem 2 (PS2) as well as changes in photosynthesis in response to abiotic stresses [63]. Since phenotypic expressions are responses of the environmental, genotypic and their interaction effects, subjecting progenies to the same environmental conditions may be an efficient way of assessing the existence of genetic variability for the fluorescence parameters.

When we analyzed the *Fo* (amplitude of 6921 to 7883 relative units) and *Fm* (37395 to 43018 relative units) (S3 Appendix) parameters, *IMC 67* x *P4B* and *IMC 67* x *SCA 24* showed the highest SCA values for *Fo* and *Fm* that corresponded to GCA of the parent *IMC 67* (p <0.01). For *Fm* the highest positive value of SCA (p<0.01) for *IMC 67* x *P4B* (p<0,01), and six other progenies (Table 7) also presented high SCA values, all of which corresponded to GCA of one of the parents *IMC 67* or *SCA 6* (p <0.01). Balasimha and contributors observed genotypic variations among cacao plants for parameters associated with chlorophyll fluorescence as promising for selection and breeding programs [12]. For the effects of negative SCA, *P4B* x *PUCALA* presented the most negative values (p <0.01), with correspondence in both parents, and another five progenies presented negative value correspondence in at least one of the parents, who had GCA (P <0.01 or p <0.05) (Fig 3, Table 7). For *Fo* there was a greater influence of the additive effects, but with complementary non-additive effects also expressive (45.6%). However, for *Fm* there was a predominance of additive effects, with 27% of non-additive effects (Table 6). In this case, improvement aimed at exploring the potential of the parents would give greater security in relation to the objectives proposed in the breeding program.

Regarding derived parameters, the results obtained for *Fv/Fm* (0.81 to 0.83) and *Fo*/*Fm* (0.18 to 0.20) which were not statistically significant and significant, respectively (S3 Appendix), were within the acceptable values, which denotes regularity and good efficiency in relation to the conversion of light energy by the PS2. Therefore, it is possible to infer that the photosynthetically active radiation did not harm the photosynthetic apparatus, or just presented photoinhibition traces [56,57,64]. The increase of *Fo*/*Fm* is related to a higher rate of plastoquinone A reduction than to oxidation of plastoquinone B and, or activity of photosystem 1 (PS1) in plants exposed to NaCl. Some authors consider the increase of *Fo*/*Fm* may be a marker of plant response to salt stress [65,66]. In this way, since the progenies were statistically different for *Fo*/*Fm*, under the conditions of the present experiment, it may be interesting to evaluate the responses of these progenies to saline stress. As for *Fv*/*Fo*, which corresponds to the potential photosynthetic activity, its decrease is related to the decrease in efficiency of the photochemical process and the electron transport chain in PS2, while its increase is related to greater capacity to convert light energy into chemical energy [67]. The observed patterns for the progenies (4.1 to 4.7) were above the minimum standards described in the literature for plants that were not subjected to stresses such as saline or related to mineral elements [56,67,68]. The progenies showed statistically different results (S3 Appendix).

For *Fv/Fm*, *P4B* x *PUCALA* showed higher SCA (p <0.01), with GCA correspondence in the *P4B* parent (p <0.01), while *IMC 67* x *PUCALA* (p <0.01) showed negative effect corresponded to the parent *IMC 67* (p <0.01) (Fig 3, Table 6), whose non-additive effects were predominant (SSsca = 70.3%) (Table 5). For *Fv*/*Fo*, *SCA 6* x *SJ 02* and *P4B* x *SJ 02* presented the most contrasting SCA values (SCA = 0.146 and -0.167, p <0.01, respectively)and corresponded to *SCA 6* (GCA = 0.091) and *P4B* (GCA = -0.087), respectively, Contrasting from what was observed for *Fo* and *Fm*, the other parameters related to chlorophyll fluorescence had a predominance of non-additive effects (Table 5). In this case, the selection of plants with characteristics of interest within or between progenies would have a greater chance of success than if the parents were used.

The performance index (*PItotal*) gives an overview of photochemical events, and, therefore, it is important in the distinction of photosynthetic behavior [69]. The amplitude of *PItotal* values (0.914 to 2.28) showed variability among the progenies for this parameter. This can be seen when we observed the results of the most contrasting progenies among them, and compared these results with the average experimental performance. Progenies of *IMC 67* x *SJ 02*, *IMC 67* x *PUCALA* and *PUCALA* x *SCA 24* were higher than progenies of *SJ 02* x *SCA 24*, *SCA 6* x *PUCALA* and *SCA 6* x *P4B* This amplitude of 76.4% among the most contrasting progenies may be useful in breeding programs that consider inclusion of parameters related to chlorophyll fluorescence [12,70]. According to Kalaji and Guo and also Kalaji and contributors, there are three essential components for vitality variations: (i) a concentration of active reaction centers; (ii) the primary photochemical quantum yield; and (iii) the strength related to the reactions in the dark, after reduction of plastoquinone A [71,72].

The evaluation of parameters related to chlorophyll fluorescence provides a diagnosis of the effects of plant-environment interactions on the performance of PS2 and, if these effects are harmful, they can reduce the functioning efficiency of this machinery [56,65,67]. The correlation between *Fv/Fo* and *Fv/Fm*, was positive and moderate (p <0.05) as previously described (Fig 5). Basically these two parameters give the same information, but with different values and amplitude. While *Fv/Fo* can show large variations with significant declines, especially with respect to stressed materials, the *Fv/Fm* values change very slowly as a response to partial photoinhibition. [73,74]. There was correlation between these two parameters (Fig 5), which remained within an acceptable range (S3 Appendix), showing absence of photoinhibition, which could be detected at different times of duration and intensity with the use of both parameters. The increase in the magnitude of the genotypic correlation to very strong, compared with its phenotypic counterpart (which was moderate), shows that there were expressive influences of random effects on the phenotypic correlation.

The correlations *of Fo*/*Fm* with *Fv*/*Fm* and *Fv/Fo* were negative, weak (*ns*) and very strong (p <0.01) respectively (Fig 5). Since these are sensitive parameters to stressful conditions, and there is variation regarding progenies potential to accumulate minerals in the leaves, among them minerals with greater stressor potential when in high concentrations, such as *Na* (S4 Appendix), it is understandable that these parameters are inversely correlated. Since *Fo*/*Fm* increases with stress, whereas under stress *Fv/Fm* and *Fv/Fo* decrease (greater photoinhibition), the absence of photoinhibition signals (acceptable values of *Fv/Fm* and *Fv/Fo* for non-stressing conditions) may be related to the absence of stress or differential tolerance of progenies to random stressors (environment-genotype interaction), since linear correlations between the parameters occurred. This can be based on the average results of *Fo*/*Fm*, *Fv/Fo* and the average differential accumulation of minerals in the progenies leaves (S3 and S4 Appendix). The magnitude of the phenotypic correlation between *Fo*/*Fm* and *Fv/Fm* was low, while its genotype counterpart was very high. This demonstrates a large number of random effects for the phenotypic correlation between *Fo*/*Fm* and *Fv/Fm* (Fig 5, S5 Appendix). Contrasting the phenotypic and genotypic correlation coefficients of *Fv/Fo* x *Fo*/*Fm*, *Fv/Fm* x *Fo*/*Fm* and *Fv/Fo* x *Fv/Fm* show that this joint analysis may serve as a refinement for studies of chlorophyll fluorescence in cacao.

The evaluated progenies showed significant statistical differences for accumulation of macro and mineral micronutrients at the leaf level, except for *Ca* and *Cu*. Vliet and Contributors reviewed the plant nutrition theme and reported that different authors define the concentration of nutrients at the leaf level as the actual absorption of nutrients by the plant and with a strong influence on crop yield [75]. The accumulation of P observed in the leaves of PUCALA x SCA 24 and IMC 67 x P4B (15.8% and 12.7% higher than the average, respectively) with the highest positive effect of SCA for IMC 67 x P4B (p <0), 01), and GCA of the P4B (p <0.05) (Fig 4, Table 9), besides the predominance of non-additive genetic effects (Table 8), indicates that a selection for P accumulation in leaves it would have greater possibility successful from the progenies.

In relation to potassium (*K*), the variations observed in the leaf tissues of the evaluated cacao progenies were 6.83 to 29.4 g kg^-1^ DM, which represented an increase of 36.2%, 32.5% and 24.7% *K* concentrations in the leaves of *SCA 6* X *IMC 67*, *IMC 67* x *SCA 24* and *IMC 67* x *PUCALA*, respectively. On the other hand, a reduction of 25.9%, 27.7% and 40.5% *K* concentration in the leaves was found for *PUCALA* x *SJ 02*, *P4B* x *SJ 02* and *SCA 6* x *P4B*, respectively (S4 Appendix). Genetic differences between genotypes of various cultivated species have been described in the literature regarding translocation and use of *K*. An increase in the efficiency of uptake, accumulation and use may have a significant effect on reduction of fertilizer use [76,77]. The progeny *SCA 6* x *IMC 67*, with a higher positive effect of SCA (p <0.05) for the accumulation of *K* in leaves, presented GCA correspondence in both parents (p <0.05) (Fig 4, Table 8), and the sums of squares show higher additive effects (SSgca = 58.3%), but also with sizable non-additive effects (SSsca = 41.7%) (Table 7). Together, this information suggests that both the use of parents and the selection within progenies gives good success chances on the improvement for this trait.

In the case of *Na*, the most expressive genic effects were non-additives, as the percentage of SSsca that explains the general variation was of the order of 76.8%. Some progenies had significant SCA values for this parameter, such as *SCA 6* x *SJ 02*, with the highest positive values (p <0.01), in contrast to *P4B* x *SJ 02* and *SCA 6* x *SCA 24* (p <0.05) (Table 9). These results corroborate the possibility of using progenies in selection stages, either for obtaining plants that accumulate more leaf *Na*, without serious phytotoxicity effects, or even in saline soils have lower *Na* accumulated in the aerial part and do not suffer damages that cause significant yield losses. In addition to well-known *Na* functions in C4 and C*A*M metabolism plants, such as regeneration of phosphoenolpyruvate, this element is chemically similar to *K*, and can, even in C3 plants, substitute *K* as a cofactor in some enzymes and have a relationship with stomata opening and closure and cellular expansion [78,79]. Gattward and contributors observed synergism between *Na* and *K*, in the photosynthetic process, water use efficiency and even in the nutrition of cacao plants [80].

Regarding leaf accumulation of *N*, the amplitude among progenies was of 15.7 to 23.4 g kg^-1^ DM for *SCA 6* x *P4B* (reduction of 19.1%) and *PUCALA* x *SJ 02* (increase of 7.5%) (S4 Appendix) when compared to the experimental average. Daymond and contributors observed significant differences in leaf *N* concentration when evaluating different cacao clones, corroborated in the present study [11]. In addition, we observed a correlation between the GCA effects (p <0.01) of *SJ 02* and *P4B* parents with the SCA effects of *IMC 67* x *SJ 02* and *P4B* x *PUCALA*, respectively (Fig 4, Table 9), and, also,high non-additive effects (SSsca = 53.9%) (Table 8). Furthermore, *SCA 24* showed potential to be tested as a parent in other crosses, since it presented high GCA value and additive effects, whose favorable alleles for leaf *N* accumulation were expressive (SSgca = 46.1%) (Table 8).

The amplitude of 6.23 to 10.3 g kg^-1^ DM observed for magnesium (*Mg*) statistically discriminated progenies. When compared to the experimental average, the accumulations of *Mg* in the leaves of *SJ 02* x *SCA 24* and *P4B* x *SJ 02* were higher than the average in 9.8 and 9.0% respectively. On the other hand, *IMC 67* x *SCA 24* accumulated 18.4% less *Mg* than the experimental average (S4 Appendix). In the range found, leaf *Mg* concentrations that caused toxicity were not observed in any of the progenies (> 50 g kg^-1^ DM) [4]. Magnesium is translocated to the plant shoot through leaf transpiration, The apoplastic concentration in the aerial part is very similar to that of the xylem sap; thus, there is an influx of *Mg*^*2+*^ into the tissue cells of the aerial part, coordinated mainly by transport proteins of the MRS2 family. Once allocated inside shoot cells, *Mg* has a wide range of known functions, among which stands out its important role in photosynthesis [81]. The progeny SJ 02 x SCA 24, which showed the highest leaf Mg concentration, also showed higher SCA (p <0.01), which matched the GCA of the SJ 02 parent (p <0.05). Therefore, there is a high possibility of genetic gains through progeny selection, since there was a predominance of non-additive effects (Tables 8 and 9, Fig 4), besides being an promising progeny, because showed interesting results for K, N and Ca (S4 Appendix).

Calcium (*Ca*) has an important structural functions as well as a signaling of plant responses to environmental factors, such as stomatal opening and closure responses [82]. However, it did not significantly differentiate the progenies (Scott-Knott, p <0.05) that showed an amplitude of 7.49 to 11.6 g kg^-1^ DM. *P4B* x *PUCALA* accumulated 15.4% more *Ca* in leaves compared to the experimental average (S4 Appendix). Possibly, the experimental conditions (standardized environment), in order to evaluate the genetic effects, are related to the absence of significant statistical differences for this element. Additionally, no concentrations of *Ca* were found that could cause leaf toxicity in any progeny, which, according to White and Brown is greater than 100 g kg^-1^ DM [4]. In addition, the GCA and SCA values, broken down by parents and progenies respectively, were not statistically different by the deviation method (Fig 4, Table 9).

*A*mong the evaluated micronutrients (*Fe*, *Zn*, *Cu* and *Mn*), only *Cu* did not show statistically significant differences among progenies probably, due to the experimental coefficient of variation (10.4%). Despite this, *PUCALA* x *SCA 24* accumulated 33.8% more and *SJ 02* x *SCA 24* accumulated 20.1% less *Cu* in the leaves than the experimental average (S4 Appendix). On the other hand, the progenies behaved in a different way regarding leaf accumulation of *Fe*, which presented amplitude among progenies of 144 to 708 mg kg^-1^ DM (S4 Appendix). The leaf concentrations of *Fe* for *PUCALA* x *SCA 24*, *SCA 6* x *P4B* and *P4B* x *SCA 24* were 54.0, 49.9 and 46.0%, respectively, higher than the general average, whereas the progenies *SCA 6* x *SJ 02*, *IMC 67* x *P4B A*nd IMC67 x *PUCALA* were 29.8, 34.8 and 40.4% lower than the general mean, respectively. Regarding the combination abilities, *P4B* x *SCA 24* presented the highest SCA effect (p <0.01), which corresponded to the GCA effects of *SCA 24* and *P4B* parents (p <0.01), with a balance between additive effects (SSgca = 50.5%) and non-additive (SS*sca* = 49.5%) (Table 8). Therefore, the use of both *P4B* and *SCA 24*, as well as their progenies, which crosses involve at least one of these parents present a potential for success in improving *Fe* absorption.

Leaf accumulation of *Zn* showed amplitude among progenies of 77.3 to 175 mg kg^-1^ DM (S4 Appendix). When the progeny averages were compared to the experimental average, *IMC 67* x *P4B* and *IMC 67* x *SCA 24* accumulated 20.0 and 11.4% more *Zn*, while the progenies *IMC 67* x *SJ 02* and *SCA 6* x *P4B* accumulated 12.9 and 17.8% less *Zn* in the leaves, respectively. On the other hand, *SJ 02* x *SCA 24* had the highest positive effect of SCA (p <0.01) and *PUCALA* x *SCA 24* showed a higher negative effect of SCA for *Zn* (p <0.05). Although the parents did not differ significantly regarding the effects of GCA for *Zn* accumulation, some progenies had significant SCA values for this mineral (Fig 4, Table 9).

For *Mn* leaf accumulation, the amplitude among progenies was 591 to 1593 mg kg^-1^ DM (S4 Appendix) where P4B x PUCALA, P4B x SCA 24 and SJ 02 x SCA 24 accumulated 34.4, 23.5 and 19.2% more Mn, respectively, whereas PUCALA x SCA 24, IMC67 x PUCALA and SCA 6 x P4B accumulated 21.5, 28.0 and 39.5% less Mn in leaves, compared to the general average. Differential effects of GCA for *PUCALA*, *SJ 02* and *SCA 24* for leaf *Mn* accumulation corresponded mainly to the progenies *SCA 6* x *PUCALA*, *P4B* x *PUCALA*, *P4B* x *SCA 24* and *P4B* x *SJ 02* (p < 0.01) (Fig 4, Table 9). The most expressive effects were non-additives, since for Zn and Mn the respective SSsca’s were 90.6% and 79.6% (Table 8). These results corroborate the possibility of using these progenies in breeding selection stages, as it was said for Na, in order to select plants more tolerant to possible toxic effects or greater use efficiency.

Vliet and Colleagues have pointed out some necessary directions that can help to understand the physiology of cacao trees. Among them, they mentioned the need to understand the translocation and use of mineral nutrients, not only *NPK* and secondary macronutrients, as *Ca* and *Mg*, but micronutrients [75]. Micronutrients, besides being required at lower concentrations than macronutrients for plant growth and reproduction, are involved in several fundamental functions, such as photosynthetic electron transport (*Fe*, *Cu* and *Mn*), enzyme activations (*Mn* and *Zn*) and metalloproteins constituents (*Fe*, *Mn*, *Cu*) [83].

Interactions between mineral nutrients occur throughout the cacao life cycle [60]. In the present experiment, concentrations of P, which presented non-significant weak or moderate phenotypic correlations with most of the other mineral elements, had a negative and moderate correlation with K (r = -0.67). The negative correlations of K with Mg (high and p<0.01) and Fe (moderate and p<0.05) (Fig 5, S5 Appendix) were as observed by [78]. These authors reported that the increase of K is related to the decrease of the accumulation of P, Ca, Mg, Fe and Mn in the aerial part. As an example, we can observe the average values of accumulation of K, Mg and Fe in two progenies in the present work. When compared to the experimental average, IMC 67 x SCA 24 accumulated 32.5% more K in the leaves but accumulated 18.4% less Mg and 21.3% less Fe than the experimental average. The progeny SCA 6 x P4B accumulated 41% less K, but 5% more Mg and 49.6% more Fe than the experimental average (S4 Appendix). Also, Na correlated with Fe (not significant by the t test) and Zn (p <0.05 by the t test), with a moderate and positive correlation with the former and moderate and negative with the latter. Nitrogen showed moderate and positive correlations with Cu (ns by t test) and Mn (p <0.05 by t test). Some genetic correlations had their magnitudes increased, such as the genotype correlation between K and P, which was moderate and negative, whereas its phenotypic counterpart was low and negative, as well as the correlation of K and Fe that changed from moderate negative (phenotypic) to high negative (genotypic) (Fig 5, S5 Appendix). The direct or inverse relationships of mineral accumulation in plants are described in the literature as one of the main mechanisms that maintains cytoplasmic and vacuolar osmolarity, as well as its importance in detoxification [82].

### Correlations between parameters of different physiological groups

The relationships between physiological parameters of growth, gas exchange, chlorophyll a fluorescence emission and leaf accumulation of mineral nutrients are important tools to understand the dynamics of the joint variation of these parameters (direct or inverse phenotypic correlations), as well as the magnitude of genetic influences (genotypic correlations). Most phenotypic correlations (with rare exceptions), among the parameters that showed statistical significance by the t test, had expressive correspondences in magnitude in the genotypic correlations for the same parameters. The values of the genotypic correlations were of the same magnitudes or higher than their corresponding phenotypic correlation values (Fig 5). In practical terms, the observed phenotypic performance, based on the correlations between the parameters, showed a significant adherence of genetic values, which show less influence of random effects and higher genic effects, since these two factors combined are the origin of phenotypic correlation [84]. Therefore, we realized that the expressive phenotypic correlations did not occur by random effects, but by genetic actions.

In the context of physiological relationships, stem biomass (*SDB*) was the parameter that presented the highest number of moderate and strong expressive correlations, with 59.4 and 65.6%, respectively, for phenotypic and genotypic correlations. These were positive all those with the other biometric parameters, besides those with *Fo*/*Fm* (moderate), *VPD*_*L*_ (moderate), *iWUE* (strong), *A/Ci* (moderate) and *Mg* (moderate). However, the *SDB* correlations with *Fm*, *Fv/Fo*, *gs*, *Ci*, *gs/VPD*_*L*_, *Ci/Ca* and *Na* were moderate negative, and with *K* it was strong and negative (Fig 5, S5 Appendix). Although *Na* translocation showed a moderate and positive correlation with *Fe*, *Fv/Fm*, *Fv/Fo*, *E* and *Cu* (Fig 5, S5 Appendix), we observed that all correlations between *Na* and growth parameters were inverse, moderate or strong, that is, plants that translocate more *Na* to the leaves tend to present reduced height and biomass of their organs (Fig 5). Relationship between vigor, translocation of mineral nutrients, water use efficiency and photochemical capacity are reported for tree species [85].

For the relationships of fluorescence parameters with growth, the moderate and significant correlations observed between *Fo* x *RDB* (positive) and *Fm* x *SDB* (negative) (Fig 5), demonstrate the possibility of selecting plants for *RDB* and *SDB* vigor in early developmental stages. In general, the parameters of fluorescence and accumulation of mineral nutrients correlated with growth. For *Fo*/*Fm* the correlation were weak or moderate with *SH* (*ns*), *SD* (*ns*), *LA* (p<0.05), *LN* (p< 0.05), *RDB* (*ns*), *SDB* (p<0.05), *LDB* (*ns*) and *TDB* (*ns*) while for *Fv*/*Fo* showed negative, moderate and significant correlations with all growth parameter and presented the same genotypic correspondences (Fig 5, S5 Appendix). On the other hand, *Fv/Fm* presented negative, weak and non-significant correlations, except for *RDB* (moderate, p <0.01), which had all their genotypic correspondences increased, which demonstrates the strong influence of random environmental effects on the phenotype for this parameter. Moreover, under the experimental conditions, *Fo*/*Fm* showed a direct correlation with growth vigor and biomass accumulation, whereas, with the same group of parameters, *Fv/Fo*, *Fv/Fm* and *Na* showed inverse correlations. Moderate and positive correlations between *Fo/Fm* with several growth parameters, such as height, leaf area and dry stem biomass, and, especially, the moderate and strong negative correlations of *Fv/Fm* and *Fv/Fo* with growth parameters indicate that the parameters related to chlorophyll fluorescence could be used in the early stages of growth to select plants of reduced vigor with dwarfing potential.

*A*ccording to Lucena and colleagues, and also Rohacek there is an increase of *Fo*/*Fm* with greater plant exposure to NaCl [65,66], whereas Lichtenthaler and colleagues report that in leaves, with partial photoinhibition, *Fm*/*Fo* values tend to decrease significantly, as *Fv/Fo* [74]. In the present work, we observed moderate negative (p <0.05) correlations between *Fm*/*Fo* with *Na* and moderate positives with *Fv/Fo* (p <0.01) and *Fv/Fm* (p <0.05) (Fig 5). Pilon-Smits and colleagues reported that most studies related to *Na* in plants focus on the phytotoxicity caused by its excess, rather than its role as a beneficial or essential element, depending on the crop. In works with partial replacement of *K*^*+*^ by *Na*^*+*^ [78], however Gattward and colleagues verified that *Na*^*+*^ can act as a beneficial mineral nutrient for cacao. These same authors identified beneficial effects of *Na* for *WUE*, photosynthesis and mineral nutrition in cacao [80].

Besides *Na*, other mineral nutrients that show positive or negative correlations with fluorescence parameters were *K*, *Mg* and *Fe* (Fig 5). For example, *K* correlated weakly and positive (r = 0.46) with *Fo*, but moderate and positive (r = 0.69) with *Fm*, whereas *Mg* showed a strong negative correlation with *Fm* and moderate and positive with *Fo*/*Fm*, whereas *Fe* was strongly correlated with *Fo* and *Fm* (Fig 5, S5 Appendix). When some authors evaluated the relationships of *N*, *P* and *K* with chlorophyll fluorescence in *Caesalpinia echinata*, did not observe the influence of these minerals to fluorescence parameters such as quantum efficiency of PS2 and performance index [86]. The relationships between fluorescence parameters and mineral accumulation are described in the literature as tools to identify tolerance to salt stress [87].

Among the fluorescence parameters, only *Fm* correlated with all leaf gas exchange parameters, with correlations of moderate and strong positive values, and strong negative correlations (Fig 5). In addition, the *Fv/Fo* parameter showed a moderate negative correlation with *iWUE* (Fig 5). These results contrast with those of Daymond and contributors, who did not find statistically significant relationships between leaf gas exchange parameters and chlorophyll fluorescence in cacao genotypes [11].

When we evaluated the correlations between leaf gas exchange parameters and mineral nutrient translocation, we observed that the correlations of *K*, *Mg* and *Fe* with gas exchange parameters were moderate to strong (Fig 5). The correlation between *Mg* and *A/Ci* can be explained by the important role of *Mg* in the activation of *RUBISCO* and as a component of the chlorophyll molecule. The negative correlation between *K* and *A/Ci* may have undergone pleiotropic influences, since a strong inverse correlation was observed between *K* and *Mg* (r = -0.74), something already described by Li and contributors [88], and that in the present study also reflected in high genotypic correlation (r = -0.86) (S5 Appendix). In this way, plants of cacao that have a greater accumulation of *K* in leaves tend to decrease the accumulation of *Mg*^*2+*^ in these organs, and, consequently, to have a lower carboxylation efficiency. On the other hand, as a strong negative genotypic correlation was observed between *K* and *Fe* (r = 0.72) (Fig 5), following the same logic [88], this may have influenced the strong positive genetic correlation between *Fe* and *A/Ci* (r = 0.86). In addition, a high positive genotypic correlation was observed for *K* and *gs* (r = 0.81) (S5 Appendix). In this regard, Rengel and Damon [77] reported that genotypes with high *K* translocation capacity between organs present higher *gs*, which at least in part also explains the positive correlation of *K* with *Ci*. Other factor that may have influenced this correlation is the negative relationship of *K* with *Mg*, since the greater accumulation of *K* in leaves tends to reduce the accumulation of *Mg*, which, consequently reduces the efficiency of carboxylation and competes for the increase of *Ci*.

From the growth parameter correlations with the mineral translocation parameters, *Fe* presented moderate and negative correlations with *LN* and *RDB*, whereas *Mg* presented a moderate positive correlation with *SDB* (Fig 5). In turn, *Na* presented moderate negative correlations with most of the growth parameters, in addition to strong negative correlations with *SH* and *LA*. Therefore, plants that accumulate a greater amount of *Na* in the leaves tend to have reduced size. High concentrations of mineral elements such as *Na* may inhibit plant growth [4], with detrimental effects on photochemical efficiency. In addition, *Na* presented a moderate and positive correlation with the photosynthesis rate and strong positive correlations with *Fv/Fo* and *E* (Fig 5). As previously mentioned, the increase of *Fv/Fo* is related to the greater capacity to convert light energy to chemical energy [67]. Thus, if the decrease of *Fv/Fo* is related to the reduction of the photochemical process efficiency and the electron transport chain of PS2 [67], then it does not appear that the correlations of *Na* with growth parameters, which were moderate or strong (p <0.01 by t-test), except for *SDB*, were due to damages of the photosynthetic apparatus.

### Parental gene expression profile

Many gene expression studies of the gibberellin biosynthetic pathway have been performed in several fruit tree species and provide insight about aspects of plant growth and development, including those dwarfism or semidwarfism phenotypes related [89–92]. As could be observed in Fig 6, the relative expression of genes involved in the first two stages of gibberellin biosynthesis showed variation among the parental contrasting genotypes for height. However, the variations in the expression profiles of the CPS and KS genes (1° pathway stage), as well as KO (2° pathway stage), do not seem to endorse the phenotypic contrast of parental height. About the diterpenoids biosynthesis pathway, when Geranylgeranyl-diphosphate (GGDP) is converted into ent-copalyl-pyrophosphate, it this compound may give rise to several secondary metabolites, terpenoids or alkaloids, as well as also occurs with ent-kaurene or ent-kaurenoic acid. This is may explain the absence of relationship between the parental phenotypes and gene expression of CPS, KS and KO. On the other hand, the gene KAO 2, whose product is downstream in the pathway, when compared to the products of the other genes evaluated [93], and whose catalytic reaction on ent-kaurenoic acid give rise the first gibberellin in the pathway (GA12), was highly downregulated in the SCA 6 and Pucala genotypes and downregulated in SCA 24. Therefore, the low relative expression of KAO 2 may be related to low size of the SCA 6, SCA 24 and Pucala genotypes. Parental genotypes that were classified like of medium and high sizes had KAO 2 expression induced when compared with the calibrator, which reinforces the relation possibility.

## Conclusions

Most of the parameters evaluated show that in order to select plants with the desired performance, including progenies of low, medium and high size, none complex improvement strategy would be necessary, since were observed high and medium heritabilities.

Among the groups of parameters, growth and biomass, as well as chlorophyll fluorescence showed, in a general way, some variables with higher GCA and others with SCA, which reveals the importance of both additive and nonaddictive effects for these parameter sets. The parental genotypes SCA 6 and SCA 24 showed the highest negative combining ability to growth and biomass, and therefore, a greater accumulation of favorable additive effects to reduce vegetative vigor, whereas the parental genotype IMC67 presented high GCA for Fo and Fm. The gas exchange group showed a predominance of variables with high GCA, and consequently larger importance of the additive effects. Within this group, the P4B and IMC 67 parentals had the highest additive gene effects for water use efficiency and SJ 02 for liquid photosynthetic rate and transpiration. For the group of parameters of translocation of mineral nutrients to the leaves, SCA 6 and IMC 67 had favorable additive effects for potassium translocation, SCA 24 and P4B for iron translocation, while SCA 24 and Pucala showed favorable additive effects for copper translocation.

The phenotypic and genotypic correlations showed the existence of associations for some parameters evaluated, with larger influence of genetic factors than environmental ones. Thus, some selection indices can be elaborated for multi traits, like the selection of plants that are sources of dwarfism, present higher water use efficiency and higher magnesium accumulation in the leaves, for example.

The CPS, KS, KO and KAO2 genes showed variation in the relative expression, and therefore showed to be useful to differentiate the parents. Despite this, the expression patterns of the CPS, KS and KO genes, which were generally induced, do not match the phenotypic characterization of the parents. However, the repressed expression of KAO2 in SCA 6, SCA 24 and Pucala confirmed the low size phenotype these parents.

## Supporting information

S1 Appendix Mean values for growth and biomass parameters of the 15 progenies of cacao. (PDF)

S2 Appendix Mean values for gas exchange parameters of the 15 progenies of cacao. (PDF)

S3 Appendix Mean values for chlorophyll fluorescence parameters of the 15 progenies of cacao. (PDF)

S4 Appendix Mean values for mineral nutrient content of the 15 progenies of cacao. (PDF)

S5 Appendix Matrix of phenotypic correlations (diagonal above) and genotypic correlations (diagonal below) for growth, biomass, fluorescence, gas exchange and nutrient contente parameters. (PDF)

## References

[1] FAOSTAT. Crops production module [Internet]. 2016 [cited 13 Jun 2016]. Available: http://faostat.fao.org/site/567/DesktopDefault.aspx?PageID=567

[2] Cervantes-Martinez C, Brown JS, Schnell RJ, Phillips-Mora W, Takrama JF, Motamayor JC. Combining Ability for Disease Resistance, Yield, and Horticultural Traits of Cacao (Theobroma cacao L.) Clones. J Am Soc Hortic Sci. 2006;131: 231–241.

[3] Almeida AAF, Valle RR. Ecophysiology of the cacao tree. Brazilian J Plant Physiol. 2007;19: 425–448. doi: 10.1590/S1677-04202007000400011

[4] White PJ, Brown PH. Plant nutrition for sustainable development and global health. Ann Bot. 2010;105: 1073–1080. doi: 10.1093/aob/mcq085 20430785 10.1093/aob/mcq085 PMC2887071

[5] Balasimha D. Towards understanding the physiology of cocoa (Theobroma cacao L.). 2011;39: 1–10.

[6] Gyau A, Smoot K, Kouame C, Diby L, Kahia J, Ofori D. Farmer attitudes and intentions towards trees in cocoa (Theobroma cacao L.) farms in Côte d’Ivoire. Agrofor Syst. 2014;88: 1035–1045. doi: 10.1007/s10457-014-9677-6

[7] Arshad FM, Bala BK, Alias EF, Abdulla I. Modelling boom and bust of cocoa production systems in Malaysia. Ecol Modell. Elsevier B.V.; 2015;309–310: 22–32. doi: 10.1016/j.ecolmodel.2015.03.020

[8] Barreto MA, Santos JCS, Correa RX, Luz EDMN, Marelli J, Souza AP. Detection of genetic resistance to cocoa black pod disease caused by three Phytophthora species. Euphytica. 2015;206: 677–687. doi: 10.1007/s10681-015-1490-4

[9] Hadley P. Physiological Characterisation of Cocoa Genetic Resources. International Workshop on Cocoa Breeding Strategies. Kuala Lumpur; 1994. pp. 97–101.

[10] FRANCISCO NETO E. Genetic Parameters and genotypic Selection of Cacao in the Brazilian Amazon. Universidade Federal de Uberlândia. 2008.

[11] Daymond AJ, Tricker PJ, Hadley P. Genotypic variation in photosynthesis in cacao is correlated with stomatal conductance and leaf nitrogen. Biol Plant. 2011;55: 99–104. doi: 10.1007/s10535-011-0013-y

[12] Balasimha D, Apshara SE, Jose CT. Genotypic variations in chlorophyll fluorescence and stomatal conductance of cocoa in relation to drought tolerance. 2013;41: 40–45.

[13] Chukwu SC, Okporie EO, Onyishi GC, Ekwu LG, Nwogbaga AC, Ede N V. Application of diallel analyses in crop improvement Department of Agronomy and Ecological Management, Enugu State University of Science. Agric Biol J NORTH Am. 2016;7: 95–106. doi: 10.5251/abjna.2016.7.2.95.106

[14] Townsend T, Segura V, Chigeza G, Penfield T, Rae A, Harvey D, et al The Use of Combining Ability Analysis to Identify Elite Parents for Artemisia annua F1 Hybrid Production. PLoS One. 2013;8 doi: 10.1371/journal.pone.0061989 23626762 10.1371/journal.pone.0061989 PMC3633910

[15] Zhang X, Lv L, Lv C, Guo B, Xu R. Combining Ability of Different Agronomic Traits and Yield Components in Hybrid Barley. PLoS One. 2015;10: e0126828 doi: 10.1371/journal.pone.0126828 26061000 10.1371/journal.pone.0126828 PMC4465181

[16] Fritsche-Neto R, Miranda GV, DeLima RO, de Souza LV, da Silva J. Herança de caracteres associados à Eficiência de utilização do fósforo em milho. Pesqui Agropecu Bras. 2010;45: 465–471. doi: 10.1590/S0100-204X2010000500005

[17] Cai Q, Yu L, Yao W, Zhang Y, Wang L, Deng J, et al Correlation and combining ability analysis of physiological traits and some agronomic traits in maize. 2014;2.

[18] Panthee DR, Perkins-veazie P, Anderson C, Ibrahem R. Diallel Analysis for Lycopene Content in the Hybrids Derived from Different Colored Parents in Tomato. 2015; 1483–1492.

[19] Padi FK, Adu-Gyamfi P, Akpertey A, Arthur A, Ofori A. Differential response of cocoa (Theobroma cacao) families to field establishment stress. Plant Breed. 2013;132: 229–236. doi: 10.1111/pbr.12039

[20] dos Santos EA, Almeida A-AF de, Ahnert D, Branco MC da S, Valle RR, Baligar VC. Diallel Analysis and Growth Parameters as Selection Tools for Drought Tolerance in Young Theobroma cacao Plants. PLoS One. 2016;11: e0160647 doi: 10.1371/journal.pone.0160647 27504627 10.1371/journal.pone.0160647 PMC4978391

[21] Zeng L, Pettigrew WT. Combining ability, heritability, and genotypic correlations for lint yield and fiber quality of Upland cotton in delayed planting. F Crop Res. Elsevier B.V.; 2015;171: 176–183. doi: 10.1016/j.fcr.2014.10.004

[22] Hansche PE, Hesse C, Beutel J, Beres W, Doyle J. The commercial potential of dwarf fruit trees. Calif Agric. 1979;33: 4–6.

[23] Fumuro M. Characteristics of Dry Matter Production and Assimilate Partitioning in the Dwarf Phenotype Japanese Persimmon (Diospyros kaki L.) cv. Nishimurawase. Japan Soc Hort Sci. 1996;

[24] Rodrigues F, Pinho R, Albuquerque C, Filho E, Goulart J. Combining Ability of Inbred Lines of Sweet Corn. Bragantia. 2009;68: 75–84. doi: 10.1590/S0006-87052009000100009

[25] Souza Júnior JO de, Carmello QA de C. Forms and Doses of Urea to Fertilize Clonal Cocoa Tree Cuttings Cultivated in Substrate. Rev Bras Cienc do Solo. 2009;32: 2367–2374. doi: 10.1590/S0100-06832008000600015

[26] EMBRAPA. Manual de análises químicas de solos, plantas e fertilizantes. Brasília; 1999. p. 370.

[27] Jackson ML. Nitrogen determinations for soil and plant tissue. In: Jackson ML, editor. Soil Chemical Analysis. Prentice H. Englewood Cliffs; 1958. pp. 183–204.

[28] Livak KJ, Schmittgen TD. Analysis of relative gene expression data using real-time quantitative PCR and the 2(-Delta Delta C(T)) Method. Methods. 2001;25: 402–408. doi: 10.1006/meth.2001.1262 11846609 10.1006/meth.2001.1262

[29] Pinheiro TT, Litholdo CG, Sereno ML, Leal GA, Albuquerque PS, Figueira A. Establishing references for gene expression analyses by RT-qPCR in Theobroma cacao tissues. Genet Mol Res. 2011;10: 3291–3305. doi: 10.4238/2011.November.17.4 22095481 10.4238/2011.November.17.4

[30] Santos ESL, Cerqueira-Silva CBM, Mori GM, Ahnert D, Mello DLN, Pires JL, et al Genetic Structure and Molecular Diversity of Cacao Plants Established as Local Varieties for More than Two Centuries: The Genetic History of Cacao Plantations in Bahia, Brazil. PLoS One. 2015;10(12). Available: http://journals.plos.org/plosone/article?id = 10.1371%2Fjournal.pone.0145276 10.1371/journal.pone.0145276 PMC4682715 26675449

[31] Box GEP., Cox DR. An analysis of transformation. J R Stat Soc. 1964;26: 211–243.

[32] Shapiro SS, Wilk MB. An Analysis of Variance Test for Normality (Complete Samples). Biometrika. 1965;52: 591–611. doi: 10.1093/biomet/52.3–4.591

[33] Griffing B. Concept of General and Specific Combining Ability in Relation to Diallel Crossing Systems. Aust J Biol Sci. 1956;9: 463–493. doi: 10.1071/BI9560463

[34] Memon S, Baloch MJ, Baloch GM, Jatoi WA. Combining ability through line × tester analysis for phenological, seed yield, and oil traits in sunflower (Helianthus annuus L.). Euphytica. Springer Netherlands; 2015;204: 199–209. doi: 10.1007/s10681-015-1368-5

[35] CRUZ, C. D.; CARNEIRO PCS. Modelos biométricos aplicados ao melhoramento genético. Viçosa; 2006.

[36] Mukaka MM. Statistics corner: A guide to appropriate use of correlation coefficient in medical research. Malawi Med J. 2012;24: 69–71. doi: 10.1016/j.cmpb.2016.01.020 23638278 PMC3576830

[37] Zanandrea I, Nassi F de L, Turchetto AC, Braga EJB, Antonio JP, Bacarin Marcos Antonio. Effect of Salinity under Fluorescence Parameters in Phasoelus vulgaris. R Bras Agrociência. 2006;12: 157–161.

[38] Silva SDVM, Luz EDMN, Pires JL, Yamada MM, Santos Filho LP Dos. Parent selection for cocoa resistance to witches’-broom. Pesqui Agropecuária Bras. 2010;45: 680–685. doi: 10.1590/S0100-204X2010000700007

[39] Padi FK, Domfeh O, Takrama J, Opoku S. An evaluation of gains in breeding for resistance to the cocoa swollen shoot virus disease in Ghana. Crop Prot. Elsevier Ltd; 2013;51: 24–31. doi: 10.1016/j.cropro.2013.04.007

[40] Santos IC Dos, Almeida A-AF De, Anhert D, Conceição AS Da, Pirovani CP, Pires JL, et al Molecular, Physiological and Biochemical Responses of Theobroma cacao L. Genotypes to Soil Water Deficit. PLoS One. 2014;9: e115746 doi: 10.1371/journal.pone.0115746 25541723 10.1371/journal.pone.0115746 PMC4277404

[41] Sprague GF, Tatum LA. General vs specific combining ability in single crosses of corn. J Am Soc Agron. 1942;34: 923–932.

[42] Fanwoua J, Bairam E, Delaire M, Buck-Sorlin G. The role of branch architecture in assimilate production and partitioning: the example of apple (Malus domestica). Front Plant Sci. 2014;5: 338 doi: 10.3389/fpls.2014.00338 25071813 10.3389/fpls.2014.00338 PMC4089354

[43] Bekele FL, Kennedy a. J, David CM, Lauckner FB, Bekele I. Numerical taxonomic studies on cacao (Theobroma cacao L.) in Trinidad. Euphytica. 1994;75: 231–240. doi: 10.1007/BF00025608

[44] Efron Y, Tade E, Epaina P. A Cocoa Growth Mutant with a Dwarfing Effect as Rootstock. International Worshop on cocoa Breeding for Improved Production Systems. 2003. pp. 79–91.

[45] Zhi A, Zhang H, Zhang Z xin, sheng Tao Y, Yue B, Zheng Y lian. Conversion of the Statistical Combining Ability into a Genetic Concept. J Integr Agric. Chinese Academy of Agricultural Sciences; 2012;11: 43–52. doi: 10.1016/S1671-2927(12)60781-0

[46] Soumelidou K, NH B, John P, Barnett J. The anatomy of developing bud union and its relationship to dwarfing in apple. Ann Bot. 1994;74: 605–611. doi: 10.1006/anbo.1994.1161

[47] Engels JMM, Fassi H. Cacao Genetic Resources Conservation and Use: Results of a Survey. Proceedmgs of the International Workshop on Cocoa Breeding Strategies. Kuala Lumpur; 1994. pp. 174–178.

[48] Winson D, Cooper JP. Diallel analysis of photosynthetic rate and related leaf characters among contrasting genotypes of Lolium perene. Heredity (Edinb). 1969;24: 633–649.

[49] Baligar VC, Bunce JA, Machado RCR, Elson MK. Photosynthetic photon flux density, carbon dioxide concentration, and vapor pressure deficit effects on photosynthesis in cacao seedlings. Photosynthetica. 2008;46: 216–221. doi: 10.1007/s11099-008-0035-7

[50] Almeida AAF, Gomes FP, Araujo RP, Santos RC, Valle RR. Leaf gas exchange in species of the Theobroma genus. Photosynthetica. 2014;52: 16–21. doi: 10.1007/s11099-013-0048-8

[51] Mielke MS, Almeida AF De, Gomes FP. Photosynthetic Traits of Five Neotropical Rainforest Tree Species: Interactions between Light Response Curves and Leaf-To-Air Vapour Pressure Deficit. Brazilian Arch Biol Technol. 2005;48: 815–824.

[52] Daley PF, Raschke K, Ball JT, Berry J a. Topography of photosynthetic activity of leaves obtained from video images of chlorophyll fluorescence. Plant Physiol. 1989;90: 1233–1238. doi: 10.1104/pp.90.4.1233 16666912 10.1104/pp.90.4.1233 PMC1061872

[53] Oren R, Sperry J, Katul G, Pataki D, Ewers B, Phillips N, et al Survey and synthesis of intra-and interspecific variation in stomatal sensitivity to vapour pressure deficit. Plant Cell Environ. 1999;22: 1515–1526. doi: 10.1046/j.1365-3040.1999.00513.x

[54] Rasheed F, Dreyer E, Richard B, Brignolas F, Brendel O, Le Thiec D. Vapour pressure deficit during growth has little impact on genotypic differences of transpiration efficiency at leaf and whole-plant level: An example from Populus nigra L. Plant, Cell Environ. 2015;38: 670–684. doi: 10.1111/pce.12423 25099629 10.1111/pce.12423

[55] Klich MG. Leaf variations in Elaeagnus angustifolia related to environmental heterogeneity. Environ Exp Bot. 2000;44: 171–183. doi: 10.1016/S0098-8472(00)00056-3 11064038 10.1016/s0098-8472(00)00056-3

[56] Konrad MLF, Bezerra Da Silva JA, Furlani PR, Caruso Machado E. Trocas gasosas e fluorescência da clorofila em seis cultivares de cafeeiro sob estresse de alumínio. Bragantia. 2005;64: 339–347. doi: 10.1590/S0006-87052005000300004

[57] Tezara W, Urich R, Jaimez R, Coronel I, Araque O, Azócar C, et al ecophysiological characteristics. Bot Sci. 2016;94: 1–12. doi: 10.17129/botsci.552

[58] Long SP, Zhu XG, Naidu SL, Ort DR. Can improvement in photosynthesis increase crop yields? Plant, Cell Environ. 2006;29: 315–330. doi: 10.1111/j.1365-3040.2005.01493.x 17080588 10.1111/j.1365-3040.2005.01493.x

[59] Schulze E, Hall AE. Stomatal Responses, Water Loss and CO 2 Assimilation Rates of Plants in Contrasting Environments In: Lange OL, editor. Physiological Plant Ecology II. Springer-V. Berlin; 1982 pp. 181–230. doi: 10.1007/978-3-642-68150-9_8

[60] Massonnet C, Costes E, Rambal S, Dreyer E, Regnard JL. Stomatal regulation of photosynthesis in apple leaves: Evidence for different water-use strategies between two cultivars. Ann Bot. 2007;100: 1347–1356. doi: 10.1093/aob/mcm222 17901058 10.1093/aob/mcm222 PMC2759240

[61] Alban MKA, Apshara SE, Hebbar KB, Mathias TG, Severin A. Morpho-physiological criteria for assessment of two month old cocoa (Theobroma cacao L.) genotypes for drought tolerance. Indian J Plant Physiol. 2015;21: 23–30. doi: 10.1007/s40502-015-0195-y

[62] Edwards CE, Ewers BE, Williams DG, Xie Q, Lou P, Xu X, et al The genetic architecture of ecophysiological and circadian traits in Brassica rapa. Genetics. 2011;189: 375–390. doi: 10.1534/genetics.110.125112 21750258 10.1534/genetics.110.125112 PMC3176123

[63] Baker NR. Chlorophyll Fluorescence: A Probe of Photosynthesis In Vivo. Annu Rev Plant Biol. 2008;59: 89–113. doi: 10.1146/annurev.arplant.59.032607.092759 18444897 10.1146/annurev.arplant.59.032607.092759

[64] Maxwell K, Johnson GN. Chlorophyll fluorescence—a practical guide. J Exp Bot. 2000;51: 659–668. doi: 10.1093/jexbot/51.345.659 10938857 10.1093/jxb/51.345.659

[65] Lucena CC de, Siqueira DL de, Martinez HEP, Cecon PR. Salt stress change chlorophyll fluorescence in mango. Rev Bras Frutic. 2012;34: 1245–1255. doi: 10.1590/S0100-29452012000400034

[66] Rohacek K. Chlorophyll fluorescence parameters: the definitions, photosynthetic meaning, and mutual relationships. Photosynthetica. 2002;40: 13–29.

[67] Li G, Wan S, Zhou J, Yang Z, Qin P. Leaf chlorophyll fluorescence, hyperspectral reflectance, pigments content, malondialdehyde and proline accumulation responses of castor bean (Ricinus communis L.) seedlings to salt stress levels. Ind Crops Prod. 2010;31: 13–19. doi: 10.1016/j.indcrop.2009.07.015

[68] Willits DH, Peet MM. Measurement of chlorophyll fluorescence as a heat stress indicator in tomato: laboratory and greenhouse comparisons. J Amer Soc Hort Sci. 2001;126: 188–194. Available: http://libnts.avrdc.org.tw/scripts/minisa.dll/144/VAVLIB/VAVLIB_WEB_REPORT/SISN+9883?COMMANDSEARCH%5Cnpapers3://publication/uuid/D13D2073-E995-4932-9AF6-2EE8C3D9FDF6

[69] Hermans C, Smeyers M, Rodriguez RM, Eyletters M, Strasser RJ, Delhaye J-PJ-P. Quality assessment of urban trees: a comparative study of physiological characterisation, airborne imaging and on site fluorescence monitoring by the OJIP-test. J Plant Physiol. 2003;160: 81–90. doi: 10.1078/0176-1617-00917 12685050 10.1078/0176-1617-00917

[70] Daymond AJ, Hadley P. The effects of temperature and light integral on early vegetative growth and chlorophyll fluorescence of four contrasting genotypes of cacao (Theobroma cacao). Ann Appl Biol. 2004;145: 257–262. doi: 10.1111/j.1744-7348.2004.tb00381.x

[71] Kalaji HM, Govindjee, Bosa K, Kocielniak J, Zuk-Goaszewska K. Effects of salt stress on photosystem II efficiency and CO2 assimilation of two Syrian barley landraces. Environ Exp Bot. 2011;73: 64–72. doi: 10.1016/j.envexpbot.2010.10.009

[72] Kalaji MH, Guo P. Chlorophyll fluorescence: A useful tool in barley plant breeding programs. In: Sánchez A, Gutierrez and SJ, editors. Photochemistry Research Progress. Nova Scien. 2008. pp. 439–463.

[73] Lichtenthaler HK. Chlorophyll Fluorescence Signatures of Leaves during the Autumnal Chlorophyll Breakdown. J Plant Physiol. Gustav Fischer Verlag, Stuttgart; 1987;131: 101–110. doi: 10.1016/S0176-1617(87)80271-7

[74] Lichtenthaler HK, Buschmann C, Knapp M. How to correctly determine the different chlorophyll fluorescence parameters and the chlorophyll fluorescence decrease ratio RFd of leaves with the PAM fluorometer. Photosynthetica. 2005;43: 379–393. doi: 10.1007/s11099-005-0062-6

[75] Vliet JA van, Slingerland M, Giller KE. Mineral nutrition of cocoa: a review Mineral Nutrition of Cocoa. Wageningen University and Research Centre. Wageningen; 2015. p. 57.

[76] Fageria NK. The use of nutrients in crop plants. CRC Press Boca Raton, FL; 2009 doi: 10.1017/CBO9781107415324.004

[77] Rengel Z, Damon PM. Crops and genotypes differ in efficiency of potassium uptake and use. Physiol Plant. 2008;133: 624–636. doi: 10.1111/j.1399-3054.2008.01079.x 18397208 10.1111/j.1399-3054.2008.01079.x

[78] Pilon-Smits EA, Quinn CF, Tapken W, Malagoli M, Schiavon M. Physiological functions of beneficial elements. Curr Opin Plant Biol. 2009;12: 267–274. doi: 10.1016/j.pbi.2009.04.009 19477676 10.1016/j.pbi.2009.04.009

[79] Murata S, Kobayashi M, Matoh T, Sekiya J. Sodium Stimulates Regeneration of Phosphoenolpyruvate in Mesophyll Chloroplasts of Amaranthus-Tricolor. Plant Cell Physiol. 1992;33: 1247–1250.

[80] Gattward JN, Almeida AAF, Souza JO, Gomes FP, Kronzucker HJ. Sodium-potassium synergism in Theobroma cacao: Stimulation of photosynthesis, water-use efficiency and mineral nutrition. Physiol Plant. 2012;146: 350–362. doi: 10.1111/j.1399-3054.2012.01621.x 22443491 10.1111/j.1399-3054.2012.01621.x

[81] Gardner C, Eberhart S. Analysis and Interpretation of the Variety Cross Diallel and Related Populations Author (s): C. O. Gardner and S. A. Eberhart Published by: International Biometric Society Stable URL: http://www.jstor.org/stable/2528181. Biometrics. 1966;22: 439–452. 5970549

[82] McAinsh MR, Pittman JK. Shaping the calcium signature. New Phytol. 2009;181: 275–294. doi: 10.1111/j.1469-8137.2008.02682.x 19121028 10.1111/j.1469-8137.2008.02682.x

[83] Kirkby EA, Römheld V. Micronutrientes na fisiologia de plantas- Funções, Absorção e Mobilidade. Informações agronômicas. 2007;118: 1–24.

[84] Waitt DE, Levin D a. Genetic and phenotypic correlations in plants: a botanical test of Cheverud’s conjecture. Heredity (Edinb). 1998;80: 310–319. doi: 10.1046/j.1365-2540.1998.00298.x

[85] Xue J, Clinton PW, Davis MR, Siddiqui T, Beets PN, Leckie AC. Genotypic variation in foliar nutrient concentrations, 13C, and chlorophyll fluorescence in relation to tree growth of radiata pine clones in a serpentine soil. J Plant Nutr Soil Sci. 2013;176: 724–733. doi: 10.1002/jpln.201200272

[86] Geraldo Rogério Faustini Cuzzuol, Emerson Campos Canal, Vinícius Novo Gama e Zanetti LV. Relationships of N, P, and K with the chlorophyll fluorescence, leaf nutrient accumulation, and soluble carbohydrate concentration in the stem of Caesalpinia echinata Lam. 2016;43: 151–158.

[87] Bavani MRZ, Peyvast G, Ghasemnezhad M, Forghani A. Assessment of Salt Tolerance in Pepper Using Chlorophyll Fluorescence and Mineral Compositions. Agric Conspec Sci cus. 2015;80: 153–158.

[88] Li Y-M, Elson M, Zhang D, He Z, Sicher R, Baligar V. Macro and Micro Nutrient Uptake Parameters and Use Efficiency in Cacao Genotypes as Influenced by Levels of Soil Applied K. Int J Plant Soil Sci. 2015;7: 80–90. doi: 10.9734/IJPSS/2015/17368

[89] Zhou Y, Underhill SJR. Breadfruit (Artocarpus altilis) gibberellin 20-oxidase genes: sequence variants, stem elongation and abiotic stress response. Tree Genet Genomes. 2016;11: 84 doi: 10.1007/s11295-015-0909-3 10.1016/j.plaphy.2015.11.012 26646240

[90] Kotoda N, Matsuo S, Honda I, Yano K, Shimizu T. Gibberellin 2-Oxidase Genes from Satsuma Mandarin (Citrus unshiu Marc.) Caused Late Flowering and Dwarfism in Transgenic Arabidopsis. Hortic J Preview. 2016;4: 1–11. doi: 10.2503/hortj.OKD-016

[91] Hollender CA, Hadiarto T, Srinivasan C, Scorza R, Dardick C. A brachytic dwarfism trait (dw) in peach trees is caused by a nonsense mutation within the gibberellic acid receptor PpeGID1c. New Phytol. 2016; 227–239. doi: 10.1111/nph.13772 26639453 10.1111/nph.13772

[92] Webster AD. Vigour mechanisms in dwarfing rootstocks for temperate fruit trees. Acta Hortic. 2004;658: 29–41.

[93] KEGG. Diterpenoid biosynthesis: Reference pathway. In: KEGG, Kyoto Encyclopedia of Genes and Genomes [Internet]. 2016 [cited 17 Apr 2017]. Available: http://www.kegg.jp.

