# Supplementary material for: Combining ability, heritability and genotypic relations of different physiological traits in cacao hybrids
Source: PLoS One. 2017 Jun 19;12(6):e0178790. doi: 10.1371/journal.pone.0178790 (PMC5476260; doi:10.1371/journal.pone.0178790)
Supplement: S1 Appendix — (PDF) [file pone.0178790.s001.pdf]

**S1 Appendix –** Mean values for growth and biomass parameters of the 15 progenies of cacao.

| | MEDIUM VALUES | | | | | | | | |
|---|---|---|---|---|---|---|---|---|---|
| **PROGENIES** | ***SH*** | ***SD*** | ***LA*** | ***LN*** | ***RDB*** | ***SDB*** | ***LDB*** | ***TDB*** | ***LAR*** |
| **SCA 6 x SJ02** | 87.5c | 12.4c | 4836b | 39.0c | 22.1e | 14.3b | 20.6c | 57.0d | 89.2a |
| **IMC67 x PUCALA** | 96.4e | 13.3c | 6903e | 40.2c | 22.4e | 19.9c | 29.4d | 71.7f | 102b |
| **SCA 6 x IMC 67** | 92.5d | 11.2b | 5010b | 38.2b | 19.4c | 10.7a | 18.6b | 48.7b | 106b |
| **IMC67 x SCA 24** | 84.2b | 12.0b | 4986b | 35.6b | 20.7c | 11.7a | 20.5c | 52.9c | 97.2a |
| **SCA 6 x PUCALA** | 97.4e | 12.7c | 5852c | 38.0b | 19.6c | 15.7b | 22.2c | 57.5d | 105b |
| **IMC 67 x SJ 02** | 84.3b | 13.3c | 5092b | 34.4b | 21.0c | 14.4b | 22.9c | 58.3d | 87.8a |
| **SCA 6 x SCA 24** | 109g | 11.2b | 5361c | 39.7c | 18.8c | 13.6b | 21.9c | 54.3c | 101b |
| **IMC 67 x P4B** | 102f | 14.0d | 7185e | 39.3c | 23.2e | 19.3c | 28.3d | 70.8f | 105b |
| **P4B x PUCALA** | 113g | 14.5d | 7535e | 43.4c | 21.1d | 25.1e | 28.9d | 75.2g | 101b |
| **P4B x SJ 02** | 116h | 14.2d | 6671d | 40.8c | 24.9e | 24.8e | 27.9d | 77.6g | 86.8b |
| **PUCALA x SJ 02** | 110g | 14.3d | 7242e | 42.6c | 19.4d | 23.1d | 31.8d | 74.2g | 98.6b |
| **SJ 02 x SCA 24** | 94.5d | 13.1c | 5731c | 40.9c | 20.5d | 20.7c | 22.9c | 64.1e | 95.1a |
| **P4B x SCA 24** | 103f | 13.5c | 6161d | 36.1b | 19.7d | 19.5c | 25.9d | 65.0e | 95.8a |
| **PUCALA x SCA 24** | 56.8a | 9.00a | 3714a | 26.5a | 6.60a | 11.6a | 15.5a | 33.7a | 112a |
| **SCA 6 x P4B** | 83.4b | 10.9b | 4904b | 29.9a | 12.7b | 20.8c | 20.2c | 53.6c | 92.6a |
| **General mean** | 95.3 | 12.6 | 5812 | 37.6 | 19.5 | 17.7 | 23.8 | 61.0 | 98.3 |
| **Range** | 36.0 ~ 125 | 7.8 ~ 23.4 | 1922 ~ 13392 | 19.8 ~ 78 | 4.56 ~ 45 | 3.41 ~ 40.7 | 5.78 ~ 51.0 | 26.8 ~ 117 | 29.1 ~ 255 |
| **C.V. (%)** | 2.24 | 5.1 | 6.5 | 6.4 | 3.7 | 6.9 | 2.7 | 2.2 | 8.2 |

Means followed by different letters in the same column represent statistically significant diferences (Scott-Knott, 5%).

Stem height (SH) in cm, stem diameter (SD) in mm, leaf area (LA) in cm, leaf number (LN), root dry biomass (RDB), stem dry biomass (SDB), leaf dry biomass (LDB) and total dry biomass (TDB) (all biomass parameters in $g\ kg^{-1}$ DM), and leaf area ratio (LAR). *C.V* = coefficient of variation, *Range* = Refers to the most contrasting plants among all progenies.
