# Supplementary material for: Combining ability, heritability and genotypic relations of different physiological traits in cacao hybrids
Source: PLoS One. 2017 Jun 19;12(6):e0178790. doi: 10.1371/journal.pone.0178790 (PMC5476260; doi:10.1371/journal.pone.0178790)
Supplement: S2 Appendix — (PDF) [file pone.0178790.s002.pdf]

S2 Appendix - Mean values for gas exchange parameters of the 15 progenies of cacao.

	MEDIUM VALUES									
**PROGENIES**	P_N	*gs*	*ci*	*E*	Vpd_L	*WUE*	*iWUE*	gs/Vpd_L	*Ci/Ca*	*A/Ci*
**SCA 6 x SJ02**	4.0a	0.09a	306a	0.82a	1.0f	5.3b	54.2c	0.09a	0.76a	0.01e
**IMC67 x PUCALA**	2.9b	0.06b	298a	0.53b	1.1e	6.8a	59.5c	0.06b	0.74a	0.01e
**SCA 6 x IMC 67**	2.8c	0.06c	287b	0.48b	1.0f	6.2a	58.4c	0.06c	0.72a	0.01e
**IMC67 x SCA 24**	3.3b	0.05c	275c	0.5b	1.1e	6.6a	67.8c	0.05c	0.68b	0.01e
**SCA 6 x PUCALA**	2.6d	0.04d	278c	0.48b	1.1e	5.9b	71.9b	0.04d	0.69b	0.01e
**IMC 67 x SJ 02**	3.0b	0.04d	265c	0.54b	1.1e	7.2a	86.6b	0.04d	0.67b	0.01e
**SCA 6 x SCA 24**	3.0b	0.05c	285b	0.51b	1.1e	6.5a	66.1c	0.05c	0.72a	0.01e
**IMC 67 x P4B**	2.4d	0.03e	268c	0.34c	1.1d	8.3a	85.0b	0.03e	0.68b	0.01e
**P4B x PUCALA**	2.2d	0.02f	96.8f	0.5b	2.1b	5.0b	137a	0.01g	0.37e	0.02c
**P4B x SJ 02**	3.2b	0.04d	113e	0.81a	2.1b	5.3b	136a	0.02f	0.43d	0.03b
**PUCALA x SJ 02**	3.2b	0.05d	135d	0.87a	2.1b	3.9c	75.6b	0.02f	0.51c	0.03c
**SJ 02 x SCA 24**	2.8c	0.04d	134d	0.91a	2.0c	3.2c	73.5b	0.02f	0.52c	0.02d
**P4B x SCA 24**	2.6d	0.03e	91.5f	0.52b	2.2a	6.1a	117a	0.01g	0.36e	0.04a
**PUCALA x SCA 24**	2.7c	0.05d	141d	0.89a	2.1c	3.5c	66.2c	0.02f	0.54c	0.02d
**SCA 6 x P4B**	2.5d	0.02f	76.8g	0.49b	2.2a	5.7b	106a	0.01g	0.30f	0.04b
**General mean**	2.9	0.05	203.2	0.61	1.54	5.7	84.1	0.036	0.58	0.02
**Range**	1.0 ~ 5.6	0.02 ~ 0.10	60 ~ 313	0.31 ~ 1.07	0.98 ~ 2.2	3.002 ~ 9.3	48.6 ~ 181	0.01 ~ 0.098	0.23 ~ 0.79	0.007 ~ 0.06
**C.V. (%)**	7.9	4.6	4.5	6.3	1.7	8.30	7.4	4.5	5.1	5.40

Means followed by different letters in the same column represent statistically significant diferences (Scott-Knott, 5%).

Net photosynthetic rate (P_N) [µmol(CO2) m^{-2} s^{-1}], stomatal conductance (gs) [mol(H2O) m^{-2} s^{-1}], intercellular CO2 concentrations (Ci) [µmol (CO2) mol^{-1}], transpiração (*E*), Vapour Pressure Deficit (Vpd_L) (kPa), instantaneous water-use efficiency (WUE = P_N/E) [µmol (CO2) $mmol^{-1}$ (H2O)], intrinsic water use efficiency (iWUE = P_N/gs) [µmol(CO2) mol^{-1}(H2O)], Ratio of intercellular and atmospheric CO2 molar fraction (Ci/Ca), carboxylation efficiency (*A*/*Ci*) (mol m^{-2} s^{-1}). C.V = coefficient of variation, Range = Refers to the most contrasting plants among all progenies.
