# Supplementary material for: Combining ability, heritability and genotypic relations of different physiological traits in cacao hybrids
Source: PLoS One. 2017 Jun 19;12(6):e0178790. doi: 10.1371/journal.pone.0178790 (PMC5476260; doi:10.1371/journal.pone.0178790)
Supplement: S3 Appendix — (PDF) [file pone.0178790.s003.pdf]

S3 Appendix - Mean values for chlorophyll fluorescence parameters of the 15 progenies of cacao.

	MEDIUM VALUES						
**PROGÊNIES**	***Fo***	***Fm***	***Fv***	***Fo/Fm***	***Fv/Fm***	***PI.Inst***	***Fv/Fo***
**SCA 6 x SJ02**	7443 c	41703c	34093b	0.18e	0.89a	1.74 c	4.59 a
**IMC67 x PUCALA**	7696 b	42382b	34403a	0.18d	0.81a	2.15 a	4.48 b
**SCA 6 x IMC 67**	7795 a	41786c	33811b	0.19c	0.81a	1.36 d	4.34 c
**IMC67 x SCA 24**	7836 a	42834a	34948a	0.18d	0.82a	1.83 c	4.46 b
**SCA 6 x PUCALA**	7551 c	41580c	33932b	0.18d	0.82a	1.20 d	4.51 b
**IMC 67 x SJ 02**	76222 c	42040b	34716a	0.18d	0.83a	2.24 a	4.56 a
**SCA 6 x SCA 24**	7547 c	41709c	33755b	0.18d	0.82a	1.62 c	4.47 b
**IMC 67 x P4B**	7843 a	41622c	33653b	0.19b	0.81a	1.3 d	4.29 c
**P4B x PUCALA**	7318 d	38168g	31131e	0.19a	0.82a	1.60 c	4.27 c
**P4B x SJ 02**	7538 c	38992f	31379e	0.19a	0.81a	1.67 c	4.18 d
**PUCALA x SJ 02**	7573 c	40635d	33455b	0.19c	0.82a	1.35 d	4.43 b
**SJ 02 x SCA 24**	7619 c	38905f	31811d	0.20a	0.82a	1.21 d	4.22 d
**P4B x SCA 24**	7143 e	39160f	31746d	0.18d	0.81a	1.34 d	4.45 b
**PUCALA x SCA 24**	6971 f	39187f	32362c	0.18e	0.83a	1.96 b	4.66 a
**SCA 6 x P4B**	7230 d	39704e	32601 c	0.18d	0.82a	1.04 d	4.52 b
**General mean**	7515	40694	33186	0.19	0.82	1.57	4.43
**Range**	6921 ~ 7883	37395 ~ 43018	30693 ~ 35386	0.18 ~ 0.20	0.80 ~ 0.85	0.91 ~ 2.28	4.12 ~ 4.71
**C.V. (%)**	1.2	0.9	1.2	1.4	1.3	9.80	1.5

Means followed by different letters in the same column represent statistically significant diferences (Scott-Knott, 5%).

Initial chlorophyll fluorescence (Fo), maximal chlorophyll fluorescence (Fm), variable chlorophyll fluorescence (Fm−Fo) (Fv), maximal photochemical efficiency of PSII (Fv/Fm), maximum quantum quantum yield of PS2 photochemistry (Fv/Fo), quantum yield baseline (Fo/Fm) e o performance index ou plant vitality (PI Inst). C.V = coefficient of variation, Range = Refers to the most contrasting plants among all progenies.
