# Supplementary material for: Combining ability, heritability and genotypic relations of different physiological traits in cacao hybrids
Source: PLoS One. 2017 Jun 19;12(6):e0178790. doi: 10.1371/journal.pone.0178790 (PMC5476260; doi:10.1371/journal.pone.0178790)
Supplement: S4 Appendix — (PDF) [file pone.0178790.s004.pdf]

Supplementary table 4 - Mean values for mineral nutrient content of the 15 progenies of cacao.

	Mean Values									
**Progenies**	***P***	***K***	***Na***	***N***	***Ca***	***Mg***	***Fe***	***Zn***	***Cu***	***Mn***
SCA 6* x *SJ02	3.0b	20.3a	3.07a	19.6a	9.58a	8.10b	238c	95.7b	11.2a	1063b
**IMC67 x *PUCALA***	3.1b	20.7a	2.56b	18.9b	9.25a	8.30b	202c	108a	10.0a	737c
SCA 6* x *IMC 67	3.0b	22.6a	2.48b	20.9a	9.88a	8.43b	310c	105a	9.83a	1023b
**IMC67 x *SCA 24***	3.2b	22.0a	2.70a	19.8a	8.28a	7.10c	267c	117a	10.9a	1035b
SCA 6* x *PUCALA	3.2b	17.4b	2.53b	19.6a	9.43a	8.43b	245c	105a	11.2a	988b
IMC 67* x *SJ 02	3.4a	17.2b	2.73a	21.1a	8.93a	8.78a	273c	91.5b	11.6a	1054b
SCA 6* x *SCA 24	3.5a	18.0b	2.60b	20.5a	9.00a	8.58b	276c	97.3b	9.60a	1029b
IMC 67* x *P4B	3.7a	15.0b	2.26b	18.4b	9.68a	8.45b	221c	126a	10.5a	861c
P4B* x *PUCALA	3.5a	14.1c	2.30b	20.7a	10.85a	9.00a	384b	111a	11.6a	1376a
P4B* x *SJ 02	3.3b	12.0c	2.48b	20.3a	9.78a	9.48a	351b	105a	9.15a	1217a
PUCALA* x *SJ 02	3.4a	12.3c	2.57b	21.4a	8.58a	9.00a	400b	101a	8.48a	1065b
SJ 02* x *SCA 24	3.0b	16.0b	2.83a	19.1b	9.50a	9.55a	398b	106a	8.28a	1221a
P4B* x *SCA 24	3.0b	16.1b	2.89a	20.5a	9.73a	9.08a	495a	109a	11.4a	1265a
PUCALA* x *SCA 24	3.8a	15.8b	3.30a	21.3a	9.68a	8.60b	522a	92.9b	13.9a	804c
SCA 6* x *P4B	3.2b	9.88d	2.83a	16.1c	9.43a	9.15a	508a	86.3b	7.63a	620c
**General mean**	3.3	16.6	2.7	19.9	9.4	8.7	339	105	10.4	1024
**Range**	2.6 ~ 4.2	6.8 ~ 29.4	1.8 ~ 3.8	15.7 ~ 23.4	7.49 ~ 11.6	6.23 ~ 10.3	144 ~ 708	77.3 ~ 175	4.47 ~ 15.2	591 ~ 1593
**C.V. (%)**	10.7	6.1	5.9	6.7	4.1	5.86	10.3	10.0	10.1	21.35

Means followed by different letters in the same column represent statistically significant differences (Scott-Knott, 5%).
Phosphorus (*P*), potassium (*K*), sodium (*Na*), nitrogen (*N*), calcium (*Ca*), magnesium (*Mg*) (all in g kg^{-1} DM), and iron (*Fe*), zinc (*Zn*), copper (*Cu*) e manganese (*Mn*) (all in mg/kg^{-1} DM). C.V = coefficient of variation, Range = Refers to the most contrasting plants among all progenies.
