# Supplementary material for: Combining ability, heritability and genotypic relations of different physiological traits in cacao hybrids
Source: PLoS One. 2017 Jun 19;12(6):e0178790. doi: 10.1371/journal.pone.0178790 (PMC5476260; doi:10.1371/journal.pone.0178790)
Supplement: S5 Appendix — (PDF) [file pone.0178790.s005.pdf]

Supplementary Table 5 - Matrix of phenotypic correlations (diagonal above) and genotypic correlations (diagonal below) for growth, biomass, fluorescence, gas exchange and nutrient content parameters.

	SH	*SD*	*LA*	*LN*	*RDB*	*SDB*	*LDB*	*TDB*	*Fo*	*Fm*	*Fo/Fm*	*Fv/Fm*	*Fv/Fo*	*A*	*gs*	*Ci*	*E*	*VpdL*	*WUE*	*iWUE*	*gs/VpdL*	*Ci/Ca*	*A/Ci*	*P*	*K*	*Na*	*N*	*Ca*	*Mg*	*Fe*	*Zn*	*Cu*	*Mn*
SH	1	0.77	0.83	0.86	0.72	0.65	0.78	0.84	0.32	-0.14	0.5	-0.49	-0.69	-0.08	-0.25	-0.12	-0.17	0.09	0.16	0.47	-0.18	-0.16	0.18	-0.16	-0.25	-0.75	0.09	0.18	0.35	-0.25	0.36	-0.47	0.54
SD	0.79	1	0.88	0.78	0.82	0.72	0.89	0.95	0.36	-0.11	0.41	-0.41	-0.61	-0.01	-0.23	-0.12	-0.08	0.1	0.17	0.48	-0.19	-0.16	0.14	-0.16	-0.24	-0.63	0.07	0.13	0.28	-0.3	0.48	-0.25	0.54
LA	0.84	0.91	1	0.78	0.64	0.79	0.96	0.94	0.29	-0.18	0.51	-0.45	-0.64	-0.27	-0.34	-0.19	-0.19	0.18	0.16	0.46	-0.3	-0.21	0.13	0.05	-0.32	-0.76	-0	0.24	0.3	-0.22	0.57	-0.3	0.35
LN	0.9	0.82	0.82	1	0.81	0.5	0.72	0.79	0.52	0.03	0.53	-0.47	-0.67	0.15	0.11	0.15	0	-0.13	0.04	0.11	0.13	0.16	-0.17	-0.25	0.07	-0.69	0.18	0.14	0.14	-0.48	0.42	-0.38	0.58
RDB	0.74	0.85	0.66	0.85	1	0.35	0.63	0.76	0.68	0.27	0.34	-0.64	-0.59	0.28	0.16	0.32	-0.2	-0.35	0.42	0.17	0.28	0.27	-0.19	-0.37	0.21	-0.66	0.02	0	-0.04	-0.67	0.51	-0.28	0.48
SDB	0.66	0.75	0.81	0.52	0.37	1	0.8	0.85	-0.13	-0.61	0.55	-0.21	-0.6	-0.29	-0.55	-0.65	0.14	0.64	-0.22	0.72	-0.61	-0.66	0.59	-0	-0.72	-0.4	-0.21	0.35	0.67	0.24	0.17	-0.45	0.3
LDB	0.8	0.93	1	0.76	0.65	0.83	1	0.95	0.25	-0.13	0.38	-0.33	-0.52	-0.12	-0.26	-0.2	-0.07	0.2	0.14	0.43	-0.27	-0.21	0.19	0.04	-0.35	-0.63	0.05	0.07	0.31	-0.19	0.45	-0.31	0.3
TDB	0.85	0.99	0.99	0.82	0.77	0.86	0.96	1	0.3	-0.19	0.5	-0.45	-0.67	-0.06	-0.26	-0.21	-0.05	0.2	0.13	0.52	-0.24	-0.24	0.24	-0.12	-0.35	-0.66	-0.06	0.17	0.38	-0.23	0.43	-0.41	0.42
Fo	0.33	0.37	0.3	0.55	0.69	-0.13	0.26	0.31	1	0.68	0.28	-0.44	-0.39	0.23	0.3	0.65	-0.32	-0.66	0.5	-0.36	0.44	0.62	-0.64	-0.19	0.46	-0.63	-0.07	-0.39	-0.4	-0.79	0.52	-0.35	0
Fm	-0.14	-0.11	-0.18	0.03	0.27	-0.62	-0.14	-0.2	0.69	1	-0.48	-0.08	0.38	0.42	0.63	0.91	-0.41	-0.92	0.64	-0.7	0.79	0.86	-0.79	-0.13	0.69	-0.15	-0.05	-0.63	-0.78	-0.78	0.15	0.04	-0.41
Fo/Fm	0.54	0.47	0.55	0.58	0.36	0.6	0.42	0.54	0.27	-0.5	1	-0.4	-0.92	-0.34	-0.41	-0.34	0.1	0.34	-0.21	0.4	-0.44	-0.3	0.18	0.06	-0.3	-0.57	-0.03	0.48	0.54	0.07	0.39	-0.43	0.4
Fv/Fm	-0.99	-0.99	-0.99	-0.99	-0.99	-0.44	-0.89	-0.99	-0.99	-0.1	-0.99	1	0.62	0.07	-0.06	-0.17	0.35	0.19	-0.41	-0.19	-0.13	-0.11	0.01	0.35	-0.26	0.54	0.23	-0.29	0.11	0.38	-0.69	0.18	-0.22
Fv/Fo	-0.72	-0.64	-0.67	-0.71	-0.62	-0.62	-0.54	-0.69	-0.39	0.39	-0.99	0.99	1	0.25	0.39	0.29	0	-0.27	0.05	-0.5	0.39	0.28	-0.22	0.1	0.24	0.68	0.03	-0.38	-0.44	0.06	-0.53	0.46	-0.54
A	-0.08	-0.03	-0.29	0.16	0.3	-0.3	-0.11	-0.05	0.24	0.44	-0.39	0.23	0.27	1	0.83	0.43	0.5	-0.35	-0.11	-0.46	0.7	0.49	-0.23	-0.35	0.39	0.35	0.22	-0.46	-0.33	-0.37	-0.28	-0.05	0.07
gs	-0.25	-0.24	-0.34	0.12	0.17	-0.57	-0.28	-0.27	0.31	0.64	-0.46	-0.19	0.4	0.88	1	0.73	0.26	-0.64	0.05	-0.77	0.93	0.79	-0.58	-0.3	0.73	0.3	0.18	-0.33	-0.58	-0.56	-0.13	0.16	-0.16
Ci	-0.12	-0.13	-0.19	0.16	0.33	-0.65	-0.2	-0.21	0.66	0.92	-0.37	-0.43	0.3	0.44	0.74	1	-0.34	-0.99	0.58	-0.73	0.89	0.97	-0.9	-0.09	0.8	-0.16	0.05	-0.36	-0.72	-0.88	0.2	0.19	-0.26
E	-0.17	-0.07	-0.19	-0.02	-0.22	0.15	-0.07	-0.06	-0.34	-0.43	0.08	0.88	0	0.54	0.26	-0.35	1	0.47	-0.85	-0.1	-0.08	-0.16	0.26	-0.06	-0.21	0.56	0.33	-0.07	0.36	0.37	-0.42	-0.05	0.22
VpdL	0.09	0.1	0.18	-0.13	-0.35	0.64	0.21	0.2	-0.67	-0.92	0.37	0.5	-0.28	-0.36	-0.65	-0.99	0.48	1	-0.67	0.65	-0.85	-0.93	0.87	0.1	-0.77	0.22	-0	0.34	0.72	0.88	-0.21	-0.18	0.25
WUE	0.17	0.18	0.16	0.06	0.47	-0.25	0.14	0.14	0.55	0.7	-0.21	-1.18	0.04	-0.18	0.07	0.62	-0.87	-0.72	1	-0.03	0.35	0.42	-0.39	0.06	0.32	-0.49	-0.24	-0.14	-0.45	-0.62	0.45	0.08	-0.26
iWUE	0.49	0.5	0.49	0.12	0.18	0.76	0.46	0.55	-0.39	-0.75	0.45	-0.43	-0.53	-0.55	-0.79	-0.77	-0.1	0.68	-0.09	1	-0.73	-0.81	0.73	0.11	-0.68	-0.31	-0.04	0.51	0.58	0.43	0.08	-0.09	0.45
gs/VpdL	-0.18	-0.19	-0.31	0.14	0.28	-0.62	-0.28	-0.24	0.45	0.8	-0.47	-0.35	0.4	0.72	0.94	0.9	-0.08	-0.85	0.38	-0.76	1	0.88	-0.72	-0.3	0.81	0.11	0.06	-0.3	-0.68	-0.74	-0.05	0.16	-0.2
Ci/Ca	-0.16	-0.16	-0.21	0.17	0.27	-0.66	-0.21	-0.24	0.64	0.87	-0.32	-0.26	0.3	0.5	0.8	0.98	-0.17	-0.93	0.45	-0.85	0.89	1	-0.92	-0.03	0.79	-0.07	0.16	-0.39	-0.68	-0.84	0.18	0.22	-0.23
A/Ci	0.18	0.15	0.13	-0.18	-0.19	0.59	0.19	0.24	-0.67	-0.82	0.19	0.05	-0.23	-0.25	-0.59	-0.9	0.28	0.88	-0.44	0.75	-0.73	-0.92	1	-0.17	-0.7	0.22	-0.14	0.26	0.65	0.81	-0.25	-0.26	0.23
P	-0.22	-0.26	0.02	-0.28	-0.5	0.02	0.01	-0.17	-0.25	-0.17	0.11	0.94	0.11	-0.47	-0.45	-0.12	-0.03	0.14	0.03	0.25	-0.43	-0.05	-0.22	1	-0.35	-0.07	0.18	0.08	0	0.1	0.2	0.4	-0.22
K	-0.28	-0.27	-0.35	0.09	0.24	-0.78	-0.42	-0.39	0.5	0.75	-0.33	-0.76	0.28	0.46	0.81	0.87	-0.23	-0.84	0.38	-0.77	0.89	0.86	-0.76	-0.67	1	0.05	0.24	-0.22	-0.74	-0.6	0.15	0.34	0

Na	-0.92	-0.8	-0.97	-0.85	-0.81	-0.53	-0.8	-0.82	-0.77	-0.2	-0.67	0.99	0.85	0.45	0.38	-0.19	0.71	0.27	-0.63	-0.37	0.14	-0.08	0.26	-0.13	0.07	1	0.05	-0.18	-0.03	0.48	-0.62	0.33	-0.24
N	0.1	0.09	-0.01	0.23	0.01	-0.23	0.04	-0.08	-0.08	-0.06	0	0.66	0.02	0.3	0.2	0.06	0.38	-0	-0.29	-0.01	0.06	0.18	-0.15	0.2	0.22	0.02	1	0	-0.03	0.02	-0.09	0.5	0.56
Ca	0.23	0.14	0.33	0.19	-0.05	0.47	0.06	0.2	-0.52	-0.85	0.69	-1.16	-0.53	-0.57	-0.48	-0.48	-0.1	0.43	-0.18	0.73	-0.4	-0.51	0.34	0.18	-0.36	-0.22	-0.18	1	0.44	0.25	0.12	0.22	0.32
Mg	0.39	0.29	0.34	0.13	-0.05	0.76	0.36	0.42	-0.44	-0.87	0.62	0.43	-0.5	-0.4	-0.67	-0.8	0.38	0.79	-0.53	0.68	-0.76	-0.75	0.72	0.02	-0.86	0.06	-0.05	0.51	1	0.55	-0.26	-0.39	0.28
Fe	-0.26	-0.33	-0.25	-0.51	-0.72	0.26	-0.22	-0.25	-0.86	-0.84	0.09	1.05	0.06	-0.41	-0.61	-0.94	0.4	0.94	-0.69	0.48	-0.79	-0.89	0.86	0.1	-0.72	0.63	-0.02	0.29	0.69	1	-0.4	-0.02	0.05
Zn	0.44	0.6	0.73	0.54	0.65	0.21	0.53	0.53	0.65	0.18	0.53	-0.99	-0.67	-0.29	-0.2	0.24	-0.54	-0.26	0.68	0.19	-0.07	0.22	-0.31	0.2	0.1	-0.92	-0.3	-0.14	-0.37	-0.59	1	0.06	0.16
Cu	-0.65	-0.37	-0.48	-0.46	-0.38	-0.6	-0.44	-0.56	-0.44	0.05	-0.6	0.42	0.59	-0.02	0.21	0.28	-0.07	-0.25	0.12	-0.09	0.22	0.31	-0.37	0.43	0.45	0.48	0.74	0.5	-0.53	-0.11	0.17	1	0.09
Mn	0.61	0.66	0.41	0.66	0.55	0.33	0.33	0.47	-0	-0.47	0.48	-0.3	-0.6	0.11	-0.22	-0.28	0.24	0.28	-0.27	0.55	-0.24	-0.25	0.24	-0.3	-0.02	-0.3	0.61	0.37	0.34	0.02	0.11	0.11	1

Stem height (*SH*) in cm, stem diameter (*SD*), leaf area (*LA*), leaf number (*LN*), root dry biomass (*RDB*) stem dry biomass (*SDB*), leaf dry biomass (*LDB*) and total dry biomass (*TDB*), Net photosynthetic rate (*A*), stomatal conductance (*gs*), intercellular CO_2 concentrations (*Ci*), transpiration (*E*), vapor pressure deficit (Vpd_L), instantaneous water-use efficiency (*WUE = A*/*E*), intrinsic water use efficiency (*iWUE = A*/*gs*), Ratio of intercellular and atmospheric CO_2 molar fraction (*Ci*/*Ca*), carboxylation efficiency *(A/Ci)*, *I*nitial chlorophyll fluorescence (*Fo*), maximal chlorophyll fluorescence (*Fm*), variable chlorophyll fluorescence (*Fm−Fo*) (*Fv*), maximal photochemical efficiency of PSII (*Fv/Fm*), maximum quantum yield of PS2 photochemistry (*Fv*/*Fo*), quantum yield baseline (*Fo*/*Fm*) and performance index or plant vitality (*PI Inst*), phosphorus (*P*), potassium (*K*), sodium (*Na*), nitrogen (*N*), calcium (*Ca*), magnesium (*Mg*), and iron (*Fe*), zinc (*Zn*), copper (*Cu*) e manganese (*Mn*).
